# A Prodrug Strategy to Conditionally Trap Therapeutic Payloads for Improved Tumor Retention

**DOI:** 10.1021/acscentsci.6c00185

**Published:** 2026-05-13

**Authors:** Deokhee Kang, Apurva Pandey, Garima Kumar, Abijeet Singh Mehta, Tyler C. Detomasi, Dashiell Anderson, Conner Bardine, Garrison Asper, Junyang Qi, Isha Nadig, Yifan Cui, Fiona M. Quimby, Jesse Ling, Youngho Seo, Bruce E. Cohen, Mekhail Anwar, Michael J. Evans, Charles S. Craik

**Affiliations:** † Department of Pharmaceutical Chemistry, 8785 University of California, San Francisco, San Francisco, California 94143, United States; ‡ Department of Radiology and Biomedical Imaging, 8785 University of California, San Francisco, San Francisco, California 94158, United States; § Department of Radiation Oncology, 8785 University of California, San Francisco, San Francisco, California 94158, United States; ∥ The Molecular Foundry, 1666 Lawrence Berkeley National Laboratory, Berkeley, California 94720, United States; ⊥ Department of Chemistry, University of California, Berkeley, Berkeley, California 94720, United States; # Division of Molecular Biophysics & Integrated Bioimaging, 1666 Lawrence Berkeley National Laboratory, Berkeley, California 94720, United States

## Abstract

Altered extracellular proteolysis has been exploited to selectively activate therapeutics in diseases such as cancer; however, once activated, extracellular drugs can diffuse away, limiting efficacy. We address this challenge by coupling proteolytic activation with membrane tethering to retain drugs within diseased tissue. To accomplish this, we developed “restricted interaction peptides” (RIPs), a delivery platform that leverages elevated proteolytic activity to activate membrane-interacting peptides, localizing cargos near the site of proteolysis. We demonstrate that RIPs can deliver diverse therapeutic cargos, including cytotoxins and radioisotopes. As proof of concept, we engineered “FRIP,” a RIP designed for cleavage by fibroblast activation protein (FAP), an endoprotease upregulated in solid tumors and fibrosis. Efficient P4–P4’ substrate sequences were identified and incorporated into FRIPs. Cell-based studies showed that, upon activation, the peptide adhered to membranes rapidly internalized and successfully delivered therapeutic cargos. Consistent with this, FRIPs delivering MMAE inhibited proliferation in an FAP-dependent manner. Imaging studies confirmed tumor targeting with minimal uptake in normal tissues. Finally, FRIPs delivering MMAE or Cu-67 exhibited potent antitumor effects. These findings establish membrane tethering as a strategy to enhance drug retention.

## Graphical abstract

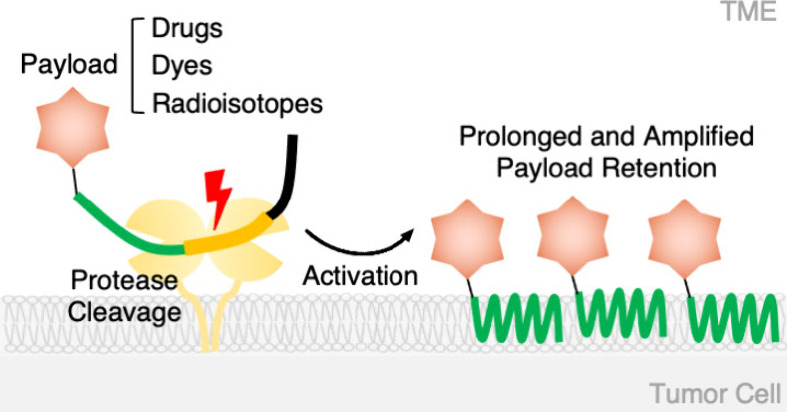

## Introduction

Chemical masking of drugs for their conditional release is a venerable strategy in oncology drug development that aims to expand the therapeutic window by activating a drug solely in the tumor while “exposing” normal tissues only to inactive drug.^1^ These so-called “pro-drug” approaches often rely on carefully positioned structural motifs that dampen the pharmacology of the parent molecule until chemical removal or modification by reactive elements enriched in the disease state (e.g., altered pH, elevated enzymatic activity, redox chemistry).^2^ With continuous drug activation at the tumor, it is expected that a high local concentration of drug will foster target engagement through passive diffusion across cancer cell membranes which ultimately results in antitumor effects. However, cancer cells often respond with powerful mechanisms to reduce tumoral drug concentration, for example, through the expression of multidrug transport proteins that promiscuously efflux small molecules from cancer cells. Thus, there is an urgent need to develop new delivery strategies that more thoughtfully shepherd the therapeutic through the complete process of release, retention, and target engagement.

To this end, we hypothesized that a prodrug strategy that couples drug activation with subsequent chemical anchoring of the activated therapeutic within the tumor could further augment tumoral drug concentration and target engagement by lengthening the residence time of the drug in the tumor. The recent renaissance in covalent therapeutics inspired this hypothesis, as the drug development community has better appreciated the impact of prolonged drug/target residence time (*k*_off_ = 0) on efficacy and therapeutic index expansion. Rather than unmasking a covalent drug per se, we envisioned a more generalized approach in which a conditionally activated tethering motif could anchor any drug within the tumor.

To test this model, we repurposed a platform we previously developed for molecular imaging termed “restricted interaction peptides” (RIP).

RIPs are approximately 30-amino-acid linear peptide composed of three modules. The N-terminal membrane binding domain (MBD), derived from the antimicrobial peptide Temporin L,^3^ facilitates membrane interaction upon proximity to cell membranes. The C-terminal peptide masking domain (PMD) prevents undesired membrane association through its electrostatic and hydrophilic properties.^4^ Between these domains lies the protease cleavage domain (PCD), engineered to be cleaved by disease-relevant proteases. Upon cleavage, the MBD undergoes a conformational shift into an amphipathic α helix, promoting targeted membrane interaction (Figure 1).^5^ This modular design enables the visualization of protease activity by delivering functional payloads, such as radioisotopes^6^ and fluorescent dyes,^4^ to target membranes. RIPs have previously been used to visualize thrombin activity in pulmonary embolism^4^ and granzyme B activity around activated immune cells in the tumor microenvironment (TME), and granzyme-mediated host immune response to viral and bacterial pathogens *in vivo* using positron emission tomography (PET).^6,7^ Notably, the granzyme B targeting RIP (GRIP B) is currently undergoing clinical trials for visualizing immune responses induced by immunotherapy (NCT06522932, NCT05888532).

**Figure 1:**
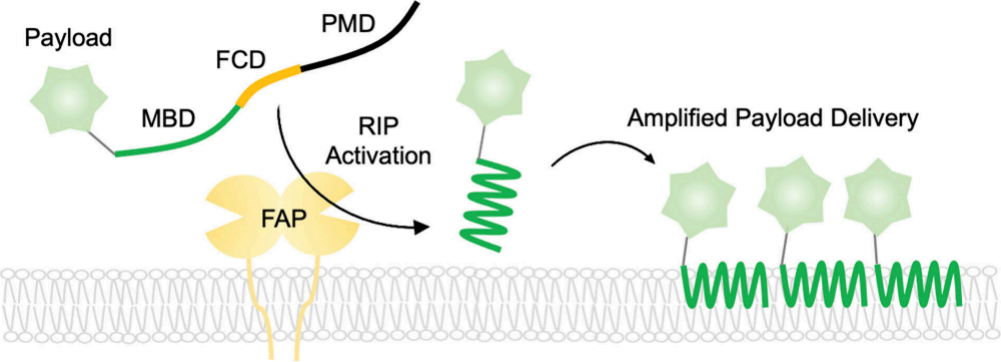
Schematic of the restricted interaction peptide. Upon FAP-mediated cleavage at the cell membrane, the amphipathic membrane-binding domain is activated and interacts with the membrane, resulting in amplified payload delivery (MBD: Membrane Binding Domain, FCD: FAP-Cleavage Domain, PMD: Peptide Masking Domain).

Fibroblast activation protein (FAP), a serine protease that cleaves after proline, has emerged as an attractive protease therapeutic target due to its highly specific overexpression in fibroblasts associated with cancer and fibrotic diseases.^8^ Since cancer-associated fibroblasts (CAFs) are generally present in all solid tumors within the TME, FAP has been deeply studied by the cancer biology and cancer drug development communities.^9^ Early efforts to target FAP therapeutically focused on stoichiometric inhibition, primarily using small molecules and antibodies to block its pro-metastatic functions.^10^ However, limitations such as protease redundancy, which complicates the development of highly effective inhibitors, have hindered the clinical efficacy of these approaches.^11,12^ As a result, a therapeutic strategy harnessing FAP enzymatic activity has emerged. Prodrugs that incorporate a FAP-cleavable peptide substrate, releasing the drug upon cleavage, are a promising approach for FAP-targeted therapy.^13,14^ Similar strategies have been explored with other proteases. For example, a pro-doxorubicin activated by prostate-specific antigen (PSA) selectively killed PSA-positive cells *in vitro* and in preclinical models of prostate cancer, further supporting the idea that exploiting proteolytic activity can achieve tumor-selective cytotoxicity.^15,16^

In this study, we show that RIPs function not only as a radioligand therapy platform but also as a peptide drug conjugate platform for targeted delivery. Because antimicrobial peptides share amphipathic and cationic properties with cell-penetrating peptides (CPPs),^17^ we observed cellular uptake and efficacy of RIP-based peptide drug conjugates, overcoming a major limitation of peptide therapeutics, namely poor cellular uptake.^18^ FAP-activated RIP variants (FRIPs) that incorporate optimized FAP-cleavable domains (FCDs) establish a protease-activated delivery platform that retains the favorable pharmacological properties of peptides while enabling protease-dependent specificity, prolonged retention, and amplified payload delivery within the TME (Figure 1).

## Results

### Design and Characterization of FRIP1

We first optimized the FCD sequence by multiplex substrate profiling using mass spectrometry (MSP-MS) (Figure 2A).^19,20^ The MSP-MS library consists of 228 rationally designed tetradecapeptide substrates with broad physicochemical diversity. All possible combinations of 20 amino acids were generated by pairing every two adjacent amino acids (XY) or every near combination (X**Y) in the library. Cys was excluded, and Met was substituted with norleucine (n) for analytic convenience. The library was incubated with recombinant human FAP and analyzed using liquid chromatography-tandem mass spectrometry (LC-MS/MS) for both time- and intensity-based measurements, generating 8-amino-acid-long optimized peptide substrate sequences. The top 5 sequences are presented in a table (Figure 2B) and illustrated as an IceLogo plot .

**Figure 2:**
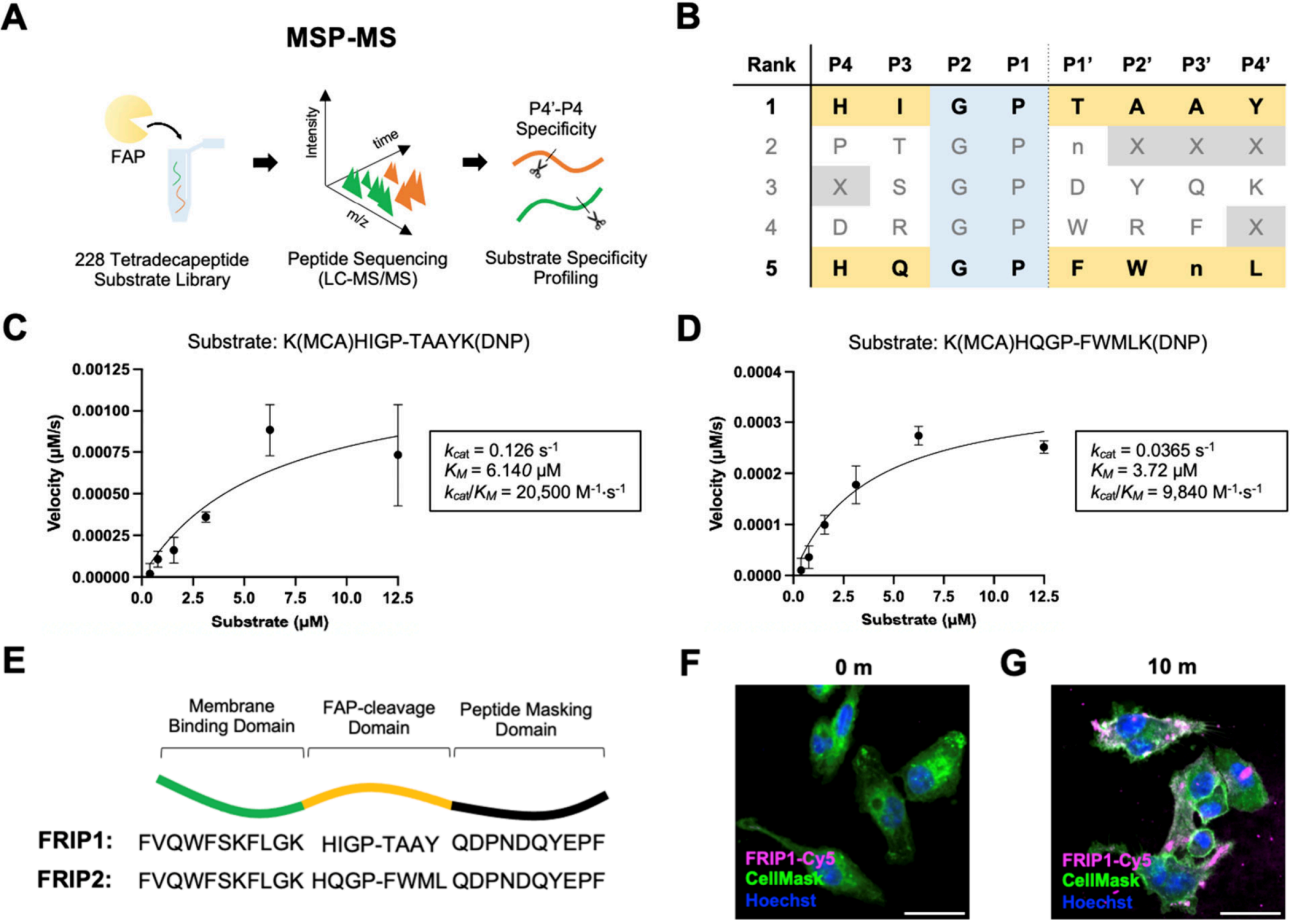
Optimization and Profiling of FRIP. (A) Schematic illustration of the MSP-MS platform. (B) Top 5 FAP substrates from the MSP-MS. (C) Enzymatic kinetics of FAP on HIGP-TAAY. (D) Enzymatic kinetics of FAP on HQGP-FWML. (E) Full peptide sequences of FRIP1 and FRIP2. (F, G) Confocal micrographs of fixed U-251 cells at 0 min (F) and at 10 m (G) after incubation with FRIP1-Cy5 (magenta: FRIP1-Cy5, green: CellMask Green, blue: Hoechst 33342, scale bar: 20 μm).

Consistent with previous reports, Pro was the most favored residue at the P1 position and Gly at the P2 position,^21^ whereas the remaining positions from P4 to P4′ tolerated broader specificity. Among the peptide substrate sequences identified by MSP-MS analysis, HIGP-TAAY (with the cleavage position denoted by a dash) showed the best substrate profile, displaying the highest fold change relative to the nonenzyme control , followed by HQGP-FWML as the second best 8-mer sequence in the list (Figure 2B). Some exopeptidase activity was also detected, as expected,^21^ further supporting the reliability of the MSP-MS analysis . Since we used an 8-mer sequence for the protease cleavage domain, HIGP-TAAY and HQGP-FWML were chosen for further investigation. To evaluate the kinetics of FAP against HIGP-TAAY and HQGP-FWML, internally quenched fluorescent (IQF) substrates were used, incorporating Lys­(MCA) and Lys­(DNP) at the N- and C-termini, respectively (Figure 2C and D). At 10 nM FAP, HIGP-TAAY exhibited a cleavage rate with a *k*_*cat*_/*K*_*M*_ of 20,500 M^–1^s^–1^, while HQGP-FWML showed a *k*_cat_/*K*_M_ of 9,840 M^–1^s^–1^. Both rates were faster than those of previously reported peptide sequences derived from collagen, a major substrate of FAP.^22^ We next inserted HIGP-TAAY between the predetermined sequences of the MBD and PMD,^4,6^ yielding the complete sequence of FRIP1 (Figure 1E).

### FRIP1 Is Activated by FAP, Resulting in Rapid Membrane Binding and Internalization of Temporin L

To investigate FRIP1 interaction with cell membranes, we imaged cells using fluorescent FRIP1 conjugates by confocal microscopy. We first screened three cancer cell lines known to endogenously express FAP and compared relative FAP expression by Western blotting . Among them, the U-251 glioblastoma cell line showed the highest FAP expression, whereas the breast cancer cell lines MDA-MB-231 and MCF7 expressed much lower levels . For confocal analysis, we selected Cy5, a far-red fluorophore previously shown to have minimal effects on RIP activity.^4^ A Cys residue was added at the N-terminus to enable site-specific conjugation (Appendix Figure 1), and FRIP1 was synthesized via solid-phase peptide synthesis (SPPS).^23^ Using maleimide–thiol chemistry, we conjugated Cy5-maleimide to Cys-FRIP1, generating the fluorescent probe FRIP1-Cy5 (Appendix Figure 2). Selective α-helix formation of Cys-FRIP1 upon proteolytic cleavage in the presence of membrane-mimicking conditions was confirmed by circular dichroism (CD) .

When 5 μM FRIP1-Cy5 was added to U-251 cells, membrane localization and internalization of FRIP1-Cy5 were observed during a 20 min time-course, as shown by both fixed cell imaging (Figure 2F and G; and live cell imaging (). At the 10 min time point, intense fluorescence signals appeared along the cell membrane, followed by internalization and cytoplasmic diffusion of FRIP1-Cy5 ). To confirm that FAP activation was required for membrane binding and internalization, a separate treatment arm was coincubated with the FAP inhibitor UAMC-1110. Co-treating U-251 cells with FRIP1-Cy5 and UAMC-1110 (1 μM, a dose that inhibits FAP but is not cytotoxic; , significantly reduced membrane interactions ). Lastly, MDA-MB-231 cells, which have low FAP expression, exhibited slower membrane accumulation of FRIP1-Cy5 compared with U-251 cells ). This slower uptake was similarly observed in intracellular fluorescence (). Notably, small Cy5-positive puncta were observed originating from cells undergoing membrane rupture , potentially influenced by probe properties. Collectively, these results show that FRIP1 is activated by endogenous FAP which results in membrane association and internalization of the Cy5 dye.

### FRIP1 Can Deliver a Cytotoxic Payload to Inhibit Cancer Cell Proliferation

We next tested if FRIP1 can deliver a therapeutic payload to inhibit the proliferation of cancer cells. MMAE, a widely used payload in the antibody-drug conjugate (ADC) field, was tested as a model system.^24^ We incorporated into FRIP a VCMMAE motif that consists of four components: a maleimidocaproyl group for maleimide–thiol click chemistry, a Val-Cit linker cleavable by cathepsins, a *p*-aminobenzyl self-immolating spacer that allows scarless removal, and the mitotoxic agent monomethyl auristatin E (MMAE). VCMMAE was tethered to FRIP1 via the N-terminal Cys to produce FRIP1-VCMMAE (Figure 3A; Appendix Figure 3).

**Figure 3:**
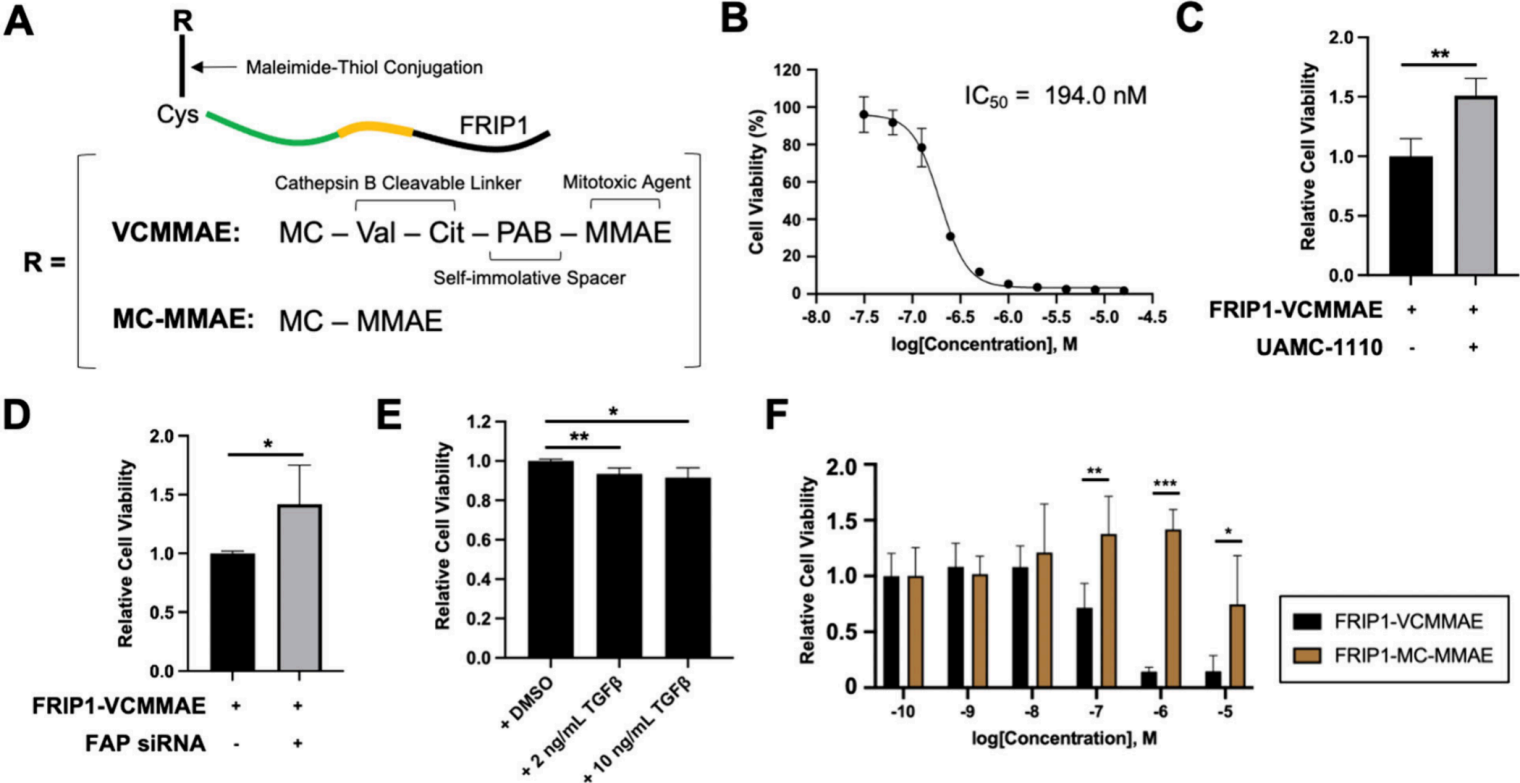
Protease-dependent toxicity of FRIP1-VCMMAE. (A) Schematic representation of the FRIP1-drug conjugates. (B) Cytotoxicity of FRIP1-VCMMAE. (C) Effect of FAP inhibitor UAMC-1110 on toxicity of 500 nM FRIP1-VCMMAE. (D) Change in toxicity of 500 nM FRIP1-VCMMAE following knockdown of endogenous FAP. (E) Effect of TGFβ cotreatment on FRIP1-VCMMAE toxicity. (F) Cytotoxicity of FRIP1-VCMMAE and FRIP1-MC-MMAE. All cells used in the experiments were U-251. (Student’s *t*-test, *: *p* < 0.05, **: *p* < 0.01, ***: *p* < 0.001; *N* = 3–5).

FRIP1-VCMMAE exhibited an IC_50_ value of 194.0 nM (Figure 3B). In contrast, Cys-FRIP1 and its cleaved form showed no cytotoxicity against U-251 cells . To serve as a control, the maleimide group of VCMMAE was quenched using excess Cys to produce Cys-VCMMAE, which showed an IC_50_ greater than 6 μM , underscoring the delivery capability of FRIP1. These findings suggest that efficient cytoplasmic delivery of MMAE via FRIP1 substantially enhances prodrug cytotoxicity. Interestingly, the FRIP1-VCMMAE cytotoxicity was comparable to that of VCMMAE alone, in U-251 cells , even with a possibility that VCMMAE may undergo nonspecific conjugation with membrane proteins, which would increase its local concentration.

The FAP-dependency of FRIP1-VCMMAE cytotoxicity was validated using multiple approaches. Co-treatment with UAMC-1110 increased cell viability by approximately 50%, confirming that inhibition of FAP reduces FRIP1-VCMMAE cytotoxicity by preventing FRIP1 activation (Figure 3C). Similarly, knockdown of endogenous FAP in U-251 cells using FAP-targeting siRNA resulted in a ∼50% increase in viability, comparable to the effect of UAMC-1110 (Figure 3C and D). Conversely, biologically enhancing FAP expression with TGFβ modestly upregulated FAP in U-251 cells^25^ and increased FRIP1-VCMMAE-induced cytotoxicity (Figure 3E). Notably, because FAP is highly efficient and capable of amplifying downstream effects even at low expression levels, and because cells were incubated with the drug conjugate for 3 days, interventions that reduced but could not completely eliminate FAP activity, still had a pronounced impact on cytotoxicity.

To confirm the intracellular, cathepsin-dependent mechanism of FRIP1-VCMMAE toxicity, we synthesized a control MMAE conjugate lacking the Val-Cit linker. This was generated by maleimide–thiol conjugation between Cys-FRIP1 and maleimidocaproyl-MMAE (MC-MMAE) (Figure 3A; Appendix Figure 4). As expected, FRIP1-MC-MMAE exhibited markedly reduced cytotoxicity in an MTT assay, confirming that intracellular release of free MMAE is essential for full therapeutic efficacy (Figure 3F).

### Molecular Imaging of FRIP1 Shows FAP-Dependent Localization to Tumors *in Vivo*

We next asked if FRIP1 can localize to tumors in animal models using noninvasive molecular imaging techniques. For deep tissue imaging, near-infrared (NIR) fluorophores are known to produce lower scatter and background compared to visible fluorophores.^26^ To image FRIP1 in mice, we synthesized an amine-reactive analog of the NIR fluorophore IR780-Cl for conjugation to the *N*-terminal amine of FRIP1 (Appendix Figure 5). The resulting conjugate has absorbance and emission peaks of 771 and 785 nm . We next employed the IQF form of FRIP1 to assess its substrate specificity against human FAP, mouse FAP, and human DPP4.^27^ Both human and mouse FAP exhibited comparable cleavage activity toward FRIP1, whereas DPP4, despite being the closest homologue, showed no detectable enzymatic activity .

We next injected mice bearing FaDu xenografts, a model of human head and neck squamous cell cancer that has been shown to recruit FAP-positive CAFs into its stromal region.^28^ After confirming FAP expression in tumors on IHC , FaDu-implanted mice were injected with FRIP1-IR780 and imaged serially out to 168 h postinjection (Figure 4A and . Tumoral uptake of the probe was visually obvious within 2 h postinjection (Figure 4B). Quantification of the NIR signal in mice showed that tumor uptake increased until 4 h postinjection and persisted to at least 24 h. No other regions of high uptake were observed, with the exception of excretory organs. To confirm tumor uptake was dependent on FAP proteolysis, a separate cohort of mice was treated with UAMC-1110 30 min prior to the FRIP1-IR780 injection. UAMC-1110 significantly reduced tumor uptake of FRIP1-IR780 at all time points measured (Figure 4C and .

**Figure 4:**
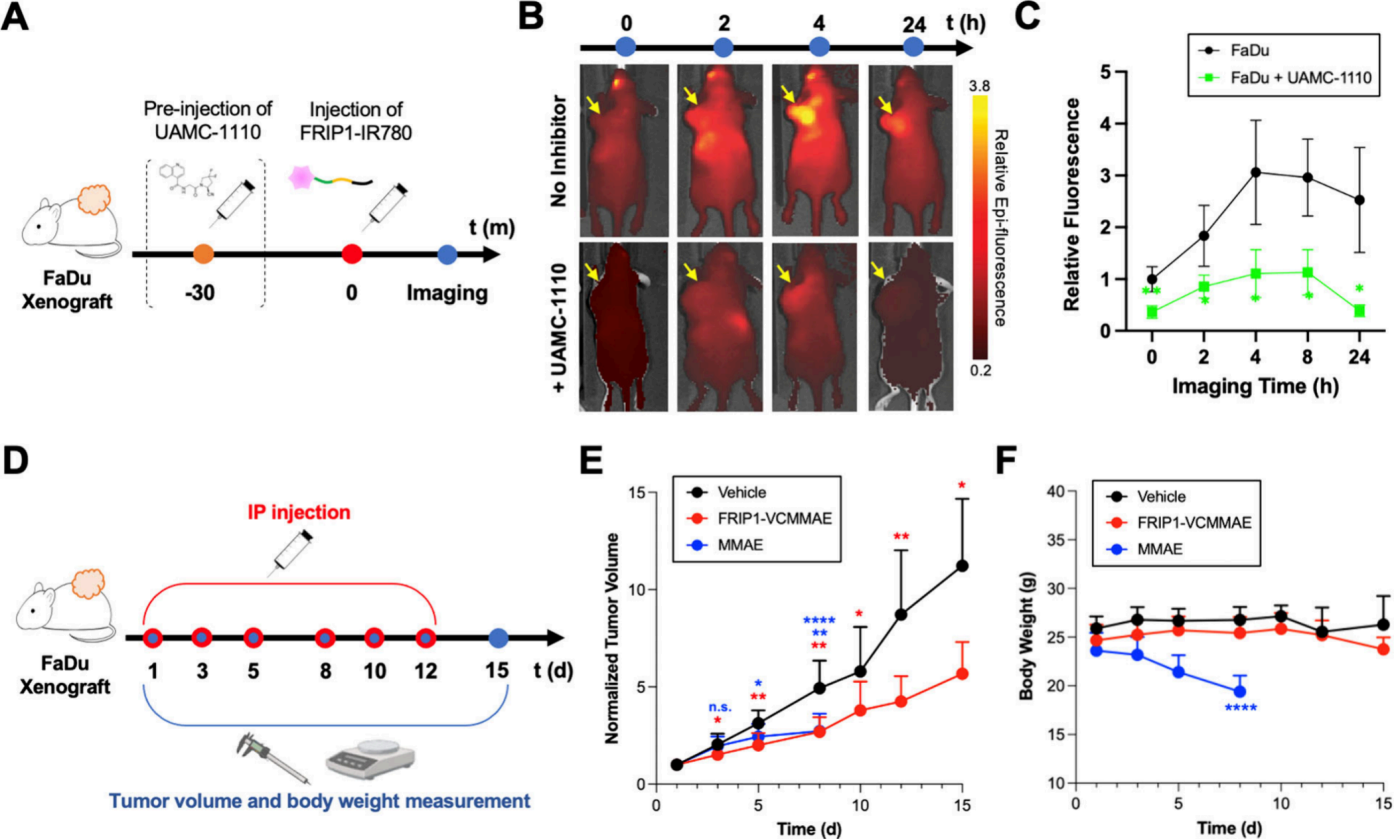
*In vivo* biodistribution analysis using FRIP1-IR780 and efficacy of FRIP1-VCMMAE. (A) Timeline of FRIP1-IR780 and UAMC-1110 injections. (B) Biodistribution of FRIP1-IR780 in FaDu-implanted mice with or without UAMC-1110. (C) Tumoral uptake of FRIP1-IR780 in FaDu-implanted mice, with and without UAMC-1110 preinjection. (Student’s *t* test, *: *p* < 0.05, **: *p* < 0.01, ***: *p* < 0.001; *N* = 3) (D) Timeline of drug injections and tumor volume and body weight measurements. (E) Tumor volume progression of vehicle-, FRIP1-VCMMAE-, and free MMAE-treated groups. (F) Body weight progression of vehicle-, FRIP1-VCMMAE-, and free MMAE-treated groups. (Student’s *t* test, *: *p* < 0.05, **: *p* < 0.01, ***: *p* < 0.001, **** indicates when all MMAE-treated mice were euthanized; *N* = 8).

### FRIP1-VCMMAE Inhibits Tumor Growth *in Vivo*

After confirming tumor uptake of FRIP1, we next tested the antitumor activity of FRIP1-VCMMAE. Separate cohorts of mice received (1) vehicle, (2) FRIP1-VCMMAE (2 mg/kg), or (3) a molar-equivalent dose of free MMAE (Figure 4D). Tumor volumes and body weights were monitored until day 15, when at least 50% of mice remained in each cohort (Figure 4E and F), and continued up to day 24 to assess antitumor and systemic effects .

FRIP1-VCMMAE induced a rapid and significant reduction in tumor size compared with both MMAE and vehicle controls (Figure 4E). This effect persisted throughout the 24-day observation period and modestly improved survival probability relative to the vehicle group . Free MMAE showed intermediate efficacy, however, FRIP1-VCMMAE consistently outperformed free MMAE until day 8 (Figure 4E; , when the MMAE cohort was euthanized due to >15% body weight loss, indicating nonspecific toxicity (Figure 4F; .

Collectively, these results demonstrate that FRIP1 efficiently delivered VCMMAE and that the released MMAE retained its cytotoxicity against FaDu tumors. No significant body weight changes were observed in the FRIP1-VCMMAE group (Figure 4F; , suggesting minimal off-target release of MMAE by endogenous proteases *in vivo.*

### FRIP Delivers Cu-64/67 to Tumors for Tumor Imaging and Therapy

We next tested if FRIP can deliver isotopic payloads to tumors for nuclear imaging and therapy. A DOTA chelator was grafted on the N-terminus of FRIP1 and FRIP2 (Appendix Figure 6 and 7). DOTA was chosen as the chelator as it is compatible with a wide range of radioisotopes for imaging and treatment (Figure 5A). Cu-64 was coupled to DOTA at high radiochemical yield and purity. Cleavage of ^64^Cu-FRIP1 and ^64^Cu-FRIP2 was confirmed by incubation with human recombinant FAP and monitored on rad-HPLC (Figure 5B; . Complete conversion to a single radioactive peak was observed within 2–12 h of incubation. The single radioactive peak coeluted with a ^64^Cu-labeled chemical standard representing cleaved FRIP1 or 2. We next performed animal imaging studies in mice bearing subcutaneous U87 MG tumors, a human glioblastoma model with endogenous expression of FAP (Figure 5C and D). PET/CT and biodistribution studies showed higher uptake of ^64^Cu-FRIP2 compared to ^64^Cu-FRIP1 (both compounds had significantly higher tumor uptake compared to the control compound ^64^Cu-GRIP B, which targets human granzyme B) (Figure 5E). The specificity of FRIP2 for FAP was further confirmed with PET/CT studies showing marginal uptake in Mia PaCa 2 or HT1080 tumors, two xenograft models with little to no FAP expression (Figure 5C and D). Region of interest analysis of longitudinal imaging data was used to determine absorbed doses in normal tissue with OLINDA (Table 1). These data showed the highest absorbed doses to be in the 0.014 mSv/mBq range and an effective dose (0.017 mSv/mBq) consistent with other diagnostic radiopharmaceuticals like ^68^Ga-FAPI-46 (0.015 mSv/mBq) and ^68^Ga-FAPI-74 (0.016 mSv/mBq). Lastly, we treated mice bearing U87 MG xenografts with 1 mCi ^67^Cu-FRIP2. A single IV dose administered at initial tumor volumes of ∼ 200 mm^3^ significantly delayed tumor growth and extended survival for the treated cohort compared to vehicle (Figure 5F).

**Figure 5:**
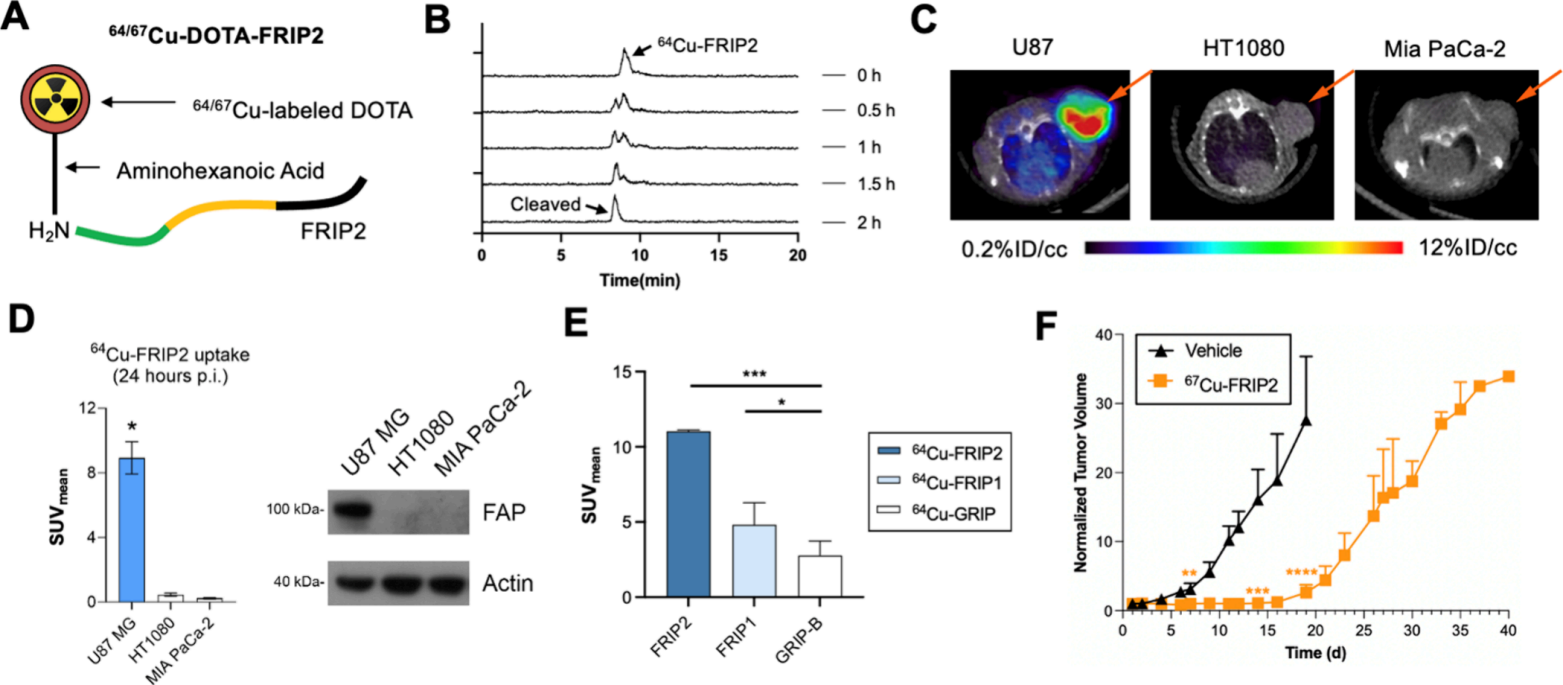
Development and validation of FRIP2, a membrane binding probe activated by FAP for targeted radiotherapy. (A) A schematic representation of FRIP2. DOTA was incorporated on the N-terminus for radiolabeling with isotopes like Cu-64 and Cu-67. (B) Representative rad-HPLC data showing the conversion of ^64^Cu-FRIP2 to a single rad peak that aligns with the retention time of the cleaved product. ^64^Cu-FRIP2 cleaved in 2 h when incubated with recombinant human FAP in buffer at 37 °C. (C) Representative PET/CT images showing high ^64^Cu-FRIP2 uptake in FAP expressing U87 MG tumors, but not in FAP null tumors HT1080 and MIA PaCa-2. (D) ROI analysis showing the significantly higher uptake of ^64^Cu-FRIP2 in U87 MG tumors at 24 h post injection. An immunoblot is shown at right depicting the relative FAP expression in the tumor models. (E) ROI analysis showing the significantly higher uptake of ^64^Cu-FRIP2 as compared to ^64^Cu-FRIP1 and ^64^Cu-GRIP-B (a granzyme B-specific RIP) in U87 MG tumors at 24 h post injection (F) Tumor volume data showing that a single dose of ^67^Cu-FRIP2 (1 mCi/mouse on day 0) significantly delays the growth of subcutaneous U87 MG, a tumor with modest and heterogeneous FAP. (Student’s *t* test, *: *p* < 0.05, **: *p* < 0.01, ***: *p* < 0.001; *N* = 4 per group).

**Table 1:** Organ-Specific Absorbed Dose Estimates for Adult Female and Male

	Absorbed Dose (mGy/MBq)	
organ	adult female (60 kg)	adult male (73 kg)
Adrenals	0.0162 ± 0.004	0.0154 ± 0.002
Brain	0.0061 ± 0.001	0.0041 ± 0.001
Breasts	0.0117 ± 0.003	0.0119 ± 0.001
Gall bladder wall	0.0171 ± 0.003	0.0166 ± 0.002
LLI wall	0.0144 ± 0.003	0.0151 ± 0.002
Small intestine	0.0140 ± 0.003	0.0152 ± 0.002
Stomach wall	0.0143 ± 0.003	0.0145 ± 0.002
ULI wall	0.0148 ± 0.004	0.0151 ± 0.002
Heart wall	0.0194 ± 0.002	0.0164 ± 0.001
Kidneys	0.0641 ± 0.018	0.0379 ± 0.011
Liver	0.0596 ± 0.018	0.0369 ± 0.009
Lungs	0.0220 ± 0.008	0.0156 ± 0.002
Muscle	0.0127 ± 0.003	0.0132 ± 0.002
Ovaries	0.0146 ± 0.003	
Pancreas	0.0159 ± 0.004	0.0156 ± 0.002
Red marrow	0.0109 ± 0.002	0.0114 ± 0.001
Osteogenic cells	0.0269 ± 0.006	0.0266 ± 0.003
Skin	0.0110 ± 0.002	0.0115 ± 0.001
Spleen	0.0142 ± 0.003	0.0142 ± 0.002
Testes		0.0133 ± 0.002
Thymus	0.0132 ± 0.003	0.0133 ± 0.002
Thyroid	0.0121 ± 0.003	0.0131 ± 0.002
Urinary bladder wall	0.0703 ± 0.017	0.0702 ± 0.013
Uterus	0.0152 ± 0.003	
Total body	0.0147 ± 0.003	0.0143 ± 0.001
Effective dose (mSv/MBq)	0.0198 ± 0.003	0.0176 ± 0.002

Tumor retention has historically been a challenge for FAP-directed radioligands, with active site-directed radioligands often achieving high SUVs in tumors but rapidly eliminating within a few hours to days postinjection. On this basis, we next tested if ^64^Cu-FRIP2 achieved greater and more persistent tumor uptake compared to FAP radioligand therapies (RLTs). We next performed longitudinal PET/CT imaging studies to compare the tumoral uptake of ^64^Cu-FRIP2 to ^64^Cu-FAPI-46, an active site-directed FAP radioligand currently in clinical trials.^29^ DOTA-FAPI-46 was coupled to Cu-64 using a similar protocol for ^64^Cu-FRIP2. The radiochemical yield and purity were >95%. ^64^Cu-FRIP2 or ^64^Cu-FAPI-46 were administered at equal doses and specific activities to mice bearing subcutaneous U87 MG tumors. Longitudinal PET studies in U87 MG tumors showed that tumoral uptake of ^64^Cu-FRIP increased from 2 to 24 h post injection and reached a SUV_mean_ of ∼ 9. In contrast, ^64^Cu-FAPI-46 levels were significantly lower over the same time interval (SUV_mean_ ∼ 1) (Figure 6A). Time-activity curves show that tumoral uptake of ^64^Cu-FRIP2 is ∼5-fold higher than tumoral uptake of ^64^Cu-FAPI-46 (Figure 6B), presumably due to the catalytic amplification of FRIP cleavage compared to stoichiometric binding of FAPI-46. Blood curves collected from a dynamic PET scan show the clearance rates of ^64^Cu-FRIP2 (*t*_1/2_ ∼ 50 s) and ^64^Cu-FAPI-46 (*t*_1/2_ ∼ 30 s) are nearly identical (Figure 6C). Lastly, a head-to-head comparison of antitumor effects showed that a single dose of 1 mCi ^67^Cu-FRIP2 more potently inhibited U87 MG tumor growth compared to ^67^Cu-FAPI-46, which is consistent with the imaging data (Figure 6D).

**Figure 6:**
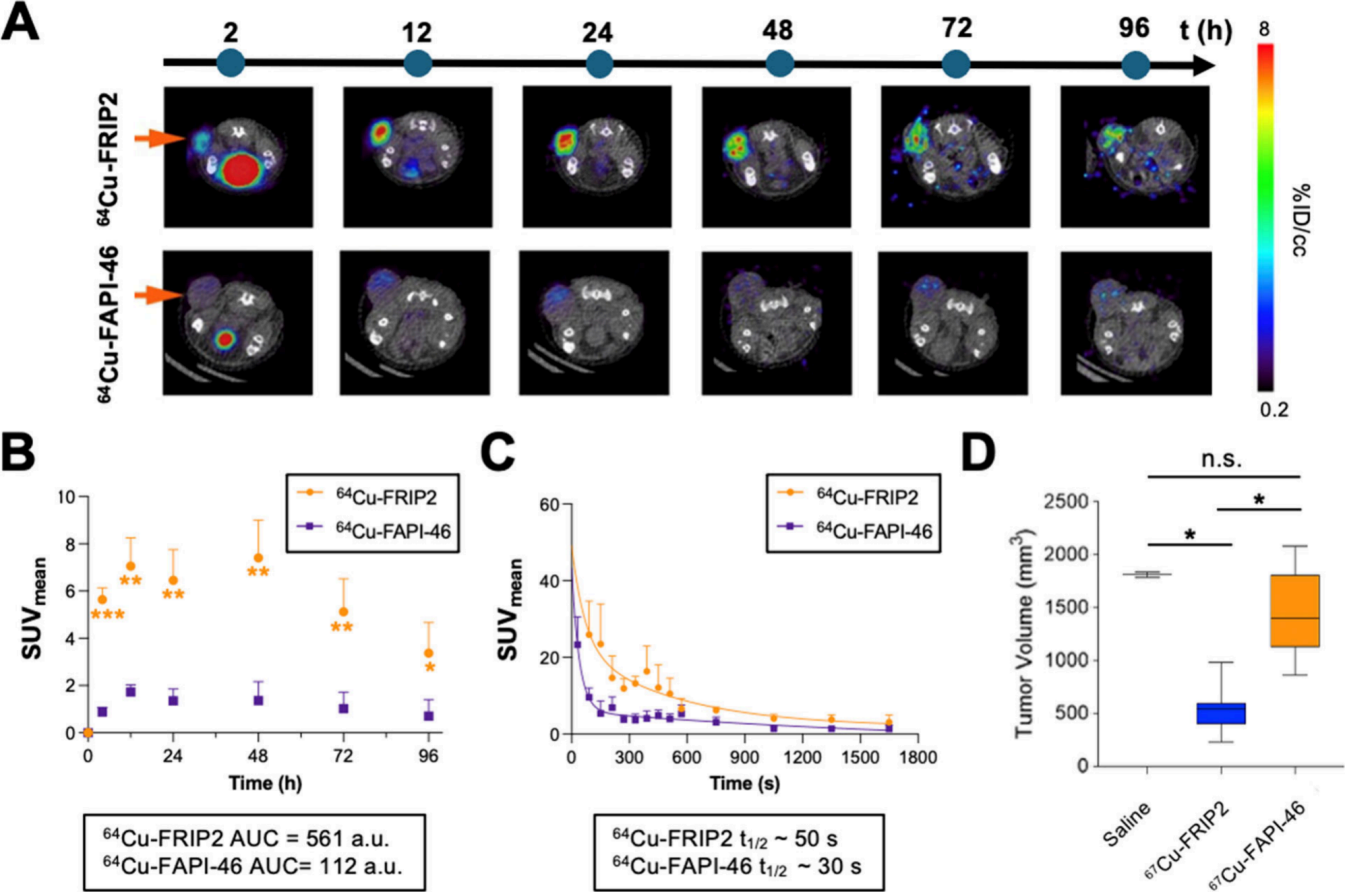
Comparative PET Imaging and pharmacokinetics of ^64^Cu-FRIP2 and ^64^Cu-FAPI-46 in U87 MG xenograft. (A) Longitudinal PET/CT scans in mice with subcutaneous U87 xenografts (orange arrow) show tumoral uptake of ^64^Cu-FRIP2 is higher and persists longer than ^64^Cu-FAPI-46. (B). Time-activity curves in U87 MG xenografts show that tumoral uptake of ^64^Cu-FRIP2 is ∼5-fold higher than tumoral uptake of ^64^Cu-FAPI-46. (C) Blood curves collected from a dynamic PET scan show the clearance rates of ^64^Cu-FRIP2 and ^64^Cu-FAPI-46 are nearly identical. (D) Day 15 tumor volume data showing that ^67^Cu-FRIP2 is more potent than ^67^Cu-FAPI-46. Both drugs were administered at 1 mCi doses on day 0 of the study. (Student’s *t* test, *: *p* < 0.05, **: *p* < 0.01, ***: *p* < 0.001; *N* = 4 per group for imaging, *N* = 8 for therapy).

## Discussion

In this report, we demonstrate that the restricted interaction peptide platform can be adapted to address an important unmet need in cancer drug development, namely precision delivery and durable retention of therapies within tumors. Using FAP as a model system for RIP activation, we show that FAP-activated RIPs (i.e., FRIPs) can be swiftly developed using an unbiased substrate discovery tool (i.e., MSP-MS). Moreover, we show that FAP activation of FRIPs results in rapid membrane binding of the free antimicrobial peptide as well as cellular internalization. Molecular imaging with IVIS or PET shows that RIPs localize to tumors in mice, with minimal binding/uptake in normal tissues lacking FAP, as expected. *In vitro* and *in vivo* studies show that FRIP can inhibit cell proliferation and tumor growth by delivering the cytotoxin MMAE within cancer cells for lysosomal activation. Lastly, we demonstrate that a single injection of ^67^Cu-FRIP potently inhibits xenograft growth, and is superior to an equivalent dose of a potent, clinical stage, FAP-targeted radioligand therapy. Collectively, these data support the further exploration of RIPs as a platform for the precision delivery of therapeutics to cancer and other diseases.

These data are timely, as FAP-targeted therapeutics are undergoing a renaissance since early studies demonstrated proof of concept nearly 20 years ago that FAP proteolysis could be leveraged for payload release within the TME.^8^ Driving this renaissance, in part, are breathtaking human PET imaging data with various active site-directed radioligands to FAP, including those bearing the venerable Gly-Pro warhead (e.g. FAPI-46 and −74) and newer binders like peptide macrocycles (e.g. FAP-2286). Generally, these data show high uptake in nearly every solid tumor type owing to FAP expression in CAFs, with low uptake in normal tissues (though high uptake can be observed in fibrotic tissues). Interestingly, radiotherapy studies using potent isotopes like Y-90, Lu-177, and Ac-225 have yet to glean equally impressive clinical responses. The meager clinical responses may be due to limited tumor retention of the FAP binders, which for unclear reasons can eliminate within a few hours of tumor binding (and generally within 72 h postinjection).

Our comparative imaging studies with FRIP and FAP RLTs show that a two-step mechanism involving proteolytic activation and membrane trapping may have special advantages compared to the classic ligand/receptor model for payload delivery. Indeed, we observed ∼5-fold increase in radiation dose delivery for FRIP compared to FAPI-46, a clinically validated FAP radiopharmaceutical, which translated into greater tumor responses in mice. The use of DOTA to chelate Cu-64/67 and the differences in PK profiles of the compounds somewhat obscures our ability to make clear comparisons between tumor-to-background ratios to rigorously test if FRIP has an expanded therapeutic index. Future studies are underway to test the impact of FRIP’s delivery mechanism on the therapeutic index compared to RLT. Meanwhile, we are completing the clinical translation of ^64^Cu-FRIP2 with first-in-human imaging studies expected to commence in 2026.

This study demonstrates the utility of FRIP1 in delivering the prodrug VCMMAE, a cathepsin-cleavable agent widely used in ADCs. Although ADCs have achieved clinical success owing to their serum stability and target specificity, they can also induce serious side effects associated with prolonged circulation and immunogenicity,^30^ which has led to growing interest in PDCs for their complementary pharmacological advantages.^31^ FRIP1 offers an attractive alternative by mitigating these risks^4^ while directly leveraging protease activity to enhance pharmacological effects through cleavage-induced membrane association, thereby improving drug efficacy and retention at tumor sites. Detailed pharmacokinetic and pharmacodynamic comparisons between FAP-targeting ADCs and FRIP1-VCMMAE will provide more precise insights into their respective advantages. Notably, the cell-permeable MMAE released from cathepsin-cleavable VCMMAE may further amplify antitumor efficacy through a bystander effect.^32^

It is unclear why FRIP2 delivered Cu-64/67 more effectively to tumors compared to FRIP1. Although FRIP2 exhibited a lower *k*_cat_ value, its stronger binding affinity may result in enhanced tumor localization. FRIP2 also outperformed FAPI-46 in radioisotope delivery, suggesting that shifting from stoichiometric binding toward exploiting proteolytic activity and subsequent membrane interactions can substantially enhance radioisotope delivery and efficacy. Indeed, comparative biodistribution data between ^64^Cu-FRIP2 and ^64^Cu-FAPI-46 clearly demonstrated that activation of the MBD effectively amplified payload delivery.

The FCD sequences identified through MSP-MS can be further optimized using structural and omics-based approaches, which have previously defined residue preferences surrounding FAP cleavage sites.^33–35^ These insights provide a framework for refining substrate sequences to improve selectivity and performance. In addition, FCDs can be adapted for other therapeutic modalities; for example, integrating FCDs into antibody-based masking strategies could combine protease-specific activation with antibody-mediated targeting.

Because individual FCD sequences can strongly influence the pharmacological properties of their conjugates, sequence-dependent behavior must be carefully considered, as illustrated by the distinct performance of FRIP2. Accordingly, each FCD sequence should be empirically validated for its intended application. Further optimization informed by residue preferences at FAP cleavage sites and large-scale analyses of natural substrates may enable the identification of sequences with improved pharmaceutical properties.

Notably, residues remaining adjacent to the MBD after proteolytic cleavage may influence membrane anchoring.^36^ For example, the more hydrophobic isoleucine at the P3 position in FRIP1 may enhance membrane association compared to the more hydrophilic glutamine in FRIP2. This interpretation is consistent with the observed differences in their membrane interaction behavior. Given that FAP is broadly expressed on cancer-associated fibroblasts, FCDs represent a potentially generalizable module for pan-cancer, FAP-activated targeting.

Computational approaches coupled with structural studies may further aid the optimization of FRIP substrates. In particular, molecular docking and dynamics simulations could provide insights into FRIP-FAP interactions and help rationalize sequence preferences observed in our screening. Such approaches may facilitate the design of substrates with improved enzymatic efficiency and membrane-targeting properties. These strategies represent a promising direction for future refinement of the FRIP platform.

Beyond the separate use of FRIP1 for chemotherapy and FRIP2 for radioligand therapy, we envision combinations of therapeutic modalities that provide multifunctionality. For instance, FRIP1 or other sequences identified through MSP-MS may serve as theranostic platforms integrating chemotherapy and radiotherapy, as exemplified by imaging-capable constructs such as ^64^Cu-FRIP1-VCMMAE, potentially yielding synergistic effects. Furthermore, inspired by the clinical success of GRIP B, we are pursuing FRIP as a potential therapeutic platform for human use.

Overall, FRIP represents a multifunctional peptide-based platform with broad utility in both therapeutic and diagnostic applications. Because the FCD module can be readily exchanged for substrates of other disease-associated proteases, this strategy may be extended to additional pathological conditions.

## Materials and Methods

No unexpected or unusually high safety hazards were encountered.

### MSP-MS

The 228-peptide library reported previously^19,37^ was incubated at a final concentration of 1 μM with 50 nM of FAP (BioLegend) or with no enzyme control (NEC) in 10 mM ammonium acetate pH 7.4. Time points were taken at 15 min, 1 h, and 4 h, followed by immediate quenching with an equal volume of 6 M Guanidinium HCl and samples were processed in the same manner that has been described previously. Two μL of each sample were injected onto a PepMAP RSLC C18 column 3 μm 100 Å, 75 μm x 15 cm (Thermo Fisher Scientific) on a 10,000 psi nanoACQUITY Ultra Performance Liquid Chromatography System (Waters) followed by a Q Exactive Plus Hybrid Quadrupole-Orbitrap (Thermo Fisher Scientific). Peptides were eluted at a flow rate of 400 nL/min using a 90 min gradient of two buffers A (0.1% formic acid in LCMS-grade water) and B (0.1% formic acid in acetonitrile). The linear gradients were as follows: 2% B for 15 min, to 25% B over 43 min, 37% B over 6 min, 40% B over 3 min, to 80% B over 3 min, to 2% B over 2 min, followed by re-equilibration at 2% B for 13 min. The resulting spectra were analyzed using MSFragger with DDA default settings using the peptide library as the reference set with decoys added. The MS data was then analyzed using the code at https://github.com/baynec2/mspms to generate fold change versus a NEC and organized by P4–P44' sequences. The top hits that contained an amino acid at all positions were then used for further analysis. The top hits were visualized using IceLogo.

### Fmoc Solid-Phase Peptide Synthesis

An internally quenched fluorogenic (IQF) form of FRIP1 and FRIP2 was synthesized from the sequences K­(MCA)­HIGPT­AAYK­(DNP) and K­(MCA)­HQGPFW­MLK­(DNP), respectively (MCA: 7-methoxycoumarin-4-acetic acid, DNP: 2-dinitrophenyl), using Fmoc solid-phase peptide synthesis on a Biotage Syro II peptide synthesizer at room temperature. The synthesis scale was at 12 μmol and started with a preloaded K­(DNP) Wang resin. Coupling reactions were performed with 4.9 equiv of O-(1*H*-6-chlorobenzotriazole-1-yl)-1,1,3,3-tetramethyluronium hexafluorophosphate (HCTU), 5 equiv of Fmoc-protected amino acids, and 20 equiv of *N*-methylmorpholine (NMM) in 500 μL of *N*,*N*-dimethylformamide (DMF) for 8 min with intermittent agitation. Every coupling was done twice, and subsequent Fmoc deprotection was executed with 500 μL of 40% 4-methylpiperidine in DMF for 10 min followed by six washes with 500 μL of DMF. The last coupling was done with K­(MCA). The peptides were cleaved from the resin using 600 μL of a cleavage cocktail solution composed of 95% trifluoroacetic acid (TFA), 2.5% water, and 2.5% triisopropylsilane for 1 h with intermittent agitation. The cleaved crude peptide product was precipitated in 50 mL of cold 1:1 diethyl ether/hexanes, dried by air, and then solubilized in DMF. The solubilized crude was purified by high-performance liquid chromatography with Agilent Pursuit 5 C18 column (5 μm bead size, 150 × 21.2 mm) on an Agilent PrepStar 218 series preparative HPLC. The mobile phases A and B were water +0.1% TFA and acetonitrile +0.1% TFA, respectively. The purified peptide product had the solvent removed under a reduced atmosphere and the dried peptides were stored in a deep freezer at −80 °C. The purity of products was assessed by liquid chromatography–mass spectrometry. Fluorescently labeled 5FAM-GRIP B was purchased from CPC Scientific at 95–98% purity.

### DOTA and Fluorophore Conjugation to FRIP

FRIP1 (FVQWFSKFL­GKHIGPTAAY­QDPNDQYEPF) and FRIP2 (FVQWFSKFL­GKHQGPFWMLQDPNDQYEPF) were synthesized using Fmoc solid-phase peptide synthesis as outlined. For maleimide–thiol conjugation, purified Cys-FRIPs were dissolved in DMF, and 3 equiv of maleimide-containing molecules along with 3 equiv of Et_3_N were added. The reaction mixture was incubated at room temperature for 30 min, then purified by HPLC and analyzed by LC-MS. For DOTA conjugation, the resin-bound FRIPs were first coupled at the N-terminus with 6-aminohexanoic acid (AHX) using standard Fmoc coupling procedures. Subsequently, 2 equiv of DOTA-SE, 5 equiv of HCTU, and 20 equiv of DIPEA were added and reacted overnight with intermittent agitation. The resulting DOTA-FRIPs were then cleaved from the resin and purified.

### Enzyme Kinetics

Proteolytic activity was measured using a BioTek H4 multimode plate reader in Corning black 384-well flat-bottom plates. Internally quenched peptides K­(MCA)­HIGPTAAYK­(DNP) or K­(MCA)­HQGPFWMLK­(DNP) were added to the reaction mixtures along with the protease of interest at a final enzyme concentration of 10 nM in 10 mM ammonium acetate buffer (pH 7.4). Michaelis–Menten kinetics were performed in triplicate at 37 °C, and fluorescence was monitored for 1 h. Initial velocities (V_0_) were calculated from the linear portion of the fluorescence curve (RFU/s) and converted to molar rates using a standard curve generated with cleaved K­(MCA). For substrate specificity experiments, 25 nM enzyme and 100 μM peptide were used under the same buffer and temperature conditions. Reactions were performed in triplicate and monitored for 1 h. When needed, protease inhibitors were added simultaneously with the AP-AFC fluorogenic substrate.

### Circular Dichroism

Spectra were obtained using a Jasco J-810 spectrometer, with samples maintained at 310 K and recorded from 250 to 200 nm at a spectral bandwidth of 1 nm and a scan rate of 50 nm min^–1^. The buffer consisted of 50 mM sodium phosphate (pH 7.4), with final peptide and SDS concentrations of 5 μM and 20 mM, respectively. Spectra were processed by subtracting the peptide-free buffer spectrum using Jasco spectra analysis software.

### Confocal Microscopy and Statistical Analysis

Human cancer cell lines (2 × 10^4^ cells per well) were seeded into 15 μ-slide 8-well chambers (ibidi) and cultured in Dulbecco’s Modified Eagle Medium (DMEM) supplemented with 10% fetal bovine serum and 1% penicillin/streptomycin. Prior to imaging, cells were washed with PBS and incubated for 15 min in Opti-MEM (Gibco) containing a 1:1000 dilution of CellMask, along with Hoechst 33342 (Invitrogen) for nuclear staining. After three PBS washes, 200 μL of phenol red-free Opti-MEM was added to each well. Time-lapse imaging was performed at 20-s intervals for a total of 20 min, beginning immediately upon addition of the peptide-dye conjugates. When applicable, inhibitors were coadministered with the conjugates. For fixed-cell imaging, cells were treated with 4% paraformaldehyde following costaining of the plasma membrane and nuclei. Images were acquired using a CSU-22 spinning disk confocal microscope at the Center for Advanced Light Microscopy (CALM), University of California, San Francisco. All fluorescence quantifications were performed using ImageJ.

### Cytotoxicity Assays

U-251 cells (5 × 10^3^ per well) were seeded into 96-well plates and cultured in DMEM supplemented with 10% fetal bovine serum and 1% penicillin/streptomycin. After a 1-day incubation to allow for cell attachment, media containing a range of concentrations of each peptide-drug conjugate were added. Following a 3-day treatment period, the supernatants were removed, and cell viability was assessed using the standard MTT assay (Abcam) performed in quintuplicate. Absorbance at 570 nm was measured with a BioTek H4 multimode plate reader. IC_50_ values were calculated using Prism 10 software.

### Animal Studies

All animal experiments were performed in accordance with the guidelines of the Institutional Animal Care and Use Committee (IACUC) at the University of California, San Francisco and were approved under protocol numbers AN203775. Six- to eight-week-old male Nu/J mice were purchased from The Jackson Laboratory and housed with free access to the water and food. Mice were inoculated with either 3 × 10^6^ FaDu or 5 × 10^6^ U87 cells in a mixture of saline and Matrigel (Corning) (v/v 1:1, 100 μL) subcutaneously into the left shoulder (imaging) or left flank (therapy). Imaging/therapy studies were initiated when the tumor size reached 100–150 mm^3^ 12–14 days post inoculation.

### Immunohistochemistry

All staining procedures performed at HistoWiz, Inc., using the Leica Bond RX automated stainer (Leica Microsystems), a Standard Operating Procedure, and a fully automated workflow. Samples were processed, embedded in paraffin, and sectioned at 4 μm. Slides were dewaxed using xylene and alcohol-based dewaxing solutions. Epitope retrieval was performed by heat-induced epitope retrieval (HIER) of the formalin-fixed, paraffin-embedded tissue using citrate-based pH 6 or EDTA-based pH 9 solution (Leica Microsystems, AR9961) for 30 min at 90 °C. Endogenous peroxidase was blocked using peroxide block buffer (Leica Microsystems). Tissue was incubated with the Anti-Fibroblast activation protein, alpha antibody [EPR20021] (Abcam, ab207178) at a 1:50 dilution for 30 min, followed by DAB rabbit secondary reagents: polymer, DAB refine and hematoxylin (Bond Polymer Refine Detection Kit, Leica Microsystems) according to the manufacturer’s protocol. The slides were dried, coverslipped (TissueTek-Prisma Coverslipper) and visualized using a Leica Aperio AT2 slide scanner (Leica Microsystems) at 40X.

### FRIP1-IR780 Administration

FRIP1-IR780 was synthesized as previously described^38^ and prepared at 50 μg/mL in 0.9% saline. All mice received a retro-orbital (RO) injection of the FRIP1-IR780. Thirty min before conjugate administration, FaDu tumor-bearing mice received an RO injection of saline (100 μL), while the remaining three FaDu tumor-bearing mice were administered the FAP inhibitor UAMC-1110 (0.125 mg of drug prepared in saline, 100 μL) via RO injection.

### *In Vivo* Imaging of FRIP1-IR780

Imaging was performed using an IVIS Spectrum system (Revvity) at excitation and emission wavelengths of 745 and 800 nm, respectively. Mice were anesthetized with 2–3% isoflurane in oxygen (2 L/min) prior to imaging. Fluorescence images were acquired at 0, 2, 4, 8, 24, 48, 72, 96, and 168 h postinjection of the FRIP1-IR780. Data were analyzed using Living Image software (version 4.8.2, Revvity). The fluorescence intensity was quantified as maximum radiant efficiency (photons/s/cm^2^/sr) for the region of interest (ROI) defining the tumor area.

### General Methods for Radioactivity Analysis

All starting materials were purchased from Acros Organics, Alfa Aesar, Sigma-Aldrich, or TCI America and used without further purification. Purity analysis of all compounds employed in biological experiments was determined by radio HPLC analysis of the radioactive complex species. Percent purity as determined by radio HPLC is reported individually for each compound; representative HPLC traces employed for purity analysis are provided within the . The reaction progress was monitored by analytical HPLC equipped with a Phenomenex Luna analytical column (C18, 100 Å, 4.6 mm × 250 mm, 10 μm) (5–95%B, A = H_2_O + 0.1% TFA, B = MeCN + 0.1% TFA over 12 min). Radiolabeling was carried out using a general protocol for all compounds reported.

### ^64^Cu Radiolabeling of DOTA-FRIP1 and DOTA-FRIP2

^64^CuCl__2__ (60 mCi, specific activity 16.5 Ci/μmol) was received from the University of Wisconsin, Madison. For radiolabeling, 5 mCi ^64^CuCl_2_ (5 μL) was diluted in 95 μL of 0.2 M NH_4_OAc buffer (pH 5.5). The reaction was carried out by adding ^64^CuCl_2_ (100 μL) to DOTA-FRIP1 or DOTA-FRIP2 (50 μg in 10 μL DMSO) in 190 μL of 0.2 M NH_4_OAc buffer (pH 5.5) at 60 °C for 30 min. Reaction progress was monitored by analytical HPLC using a Phenomenex Luna C18 column (100 Å, 4.6 mm × 250 mm, 10 μm) with a 12 min gradient from 5 to 95% B (A = H_2_O + 0.1% TFA, B = MeCN + 0.1% TFA). After 30 min, the crude reaction mixture was purified on a C18 Sep-Pak cartridge and eluted with 2 mL ethanol. Ethanol was removed at 50 °C under vacuum with a gentle stream of N_2_ to yield purified ^64^Cu-FRIP1 and ^64^Cu-FRIP2. Chelation efficiency was >95% for all constructs as determined by HPLC. For animal studies, radiolabeled peptides were formulated in 10% DMSO, 10% Tween 80, and 80% saline.

### ^67^Cu Radiolabeling of DOTA-FRIP2

^67^CuCl_2_ 20mCi was received from Idaho State University. Twenty mCi ^67^CuCl_2_ (20 μL) was diluted in NH_4_OAc buffer (80 μL, 0.2 M, pH = 5.5). Radiolabeling was performed by adding ^67^CuCl_2_ (100 μL) to a reaction mixture of DOTA-FRIP2 (200 μg in 40 μL DMSO) in NH_4_OAc buffer (160 μL, 0.2 M, pH = 5.5) at 60 °C for 30 min. The reaction progress was monitored by analytical HPLC equipped with a Phenomenex Luna analytical column (C18, 100 Å, 4.6 mm × 250 mm, 10 μm) (5–95%B, A= H_2_O + 0.1% TFA, B = MeCN + 0.1% TFA over 12 min). After 30 min, the crude reaction was purified using a C18 Sep-Pak cartridge and eluted with 2 mL of EtOH. The EtOH was then removed at 50 °C under vacuum and a gentle stream of *N*_2_(*g*) to afford clean ^67^Cu-DOTA-FRIP2. The chelation efficiency was >95% for all three constructs based on the HPLC. A formulation comprising 10% DMSO, 10% Tween 80, and 80% saline was used for animal studies.

### *In Vitro* Radioactive Cleavage Assay with FRIP1 and FRIP2

The *in vitro* cleavage of ^64^Cu-FRIP1 and ^64^Cu-FRIP2 by rhFAP was assessed by dissolving ∼ 2.5 mCi ^64^Cu-FRIP1 and ^64^Cu-FRIP2 in 300 μL of assay buffer (50 mM Tris, 1 M NaCl, 1 mg/mL BSA, pH 7.5) respectively, followed by the addition of 1.1 μg rhFAP (2.5 μL). The reaction vial was then incubated at 37 °C. The cleavage was monitored at specific time points using RAD analytical HPLC equipped with a Phenomenex Luna C18 analytical column (100 Å, 4.6 mm × 250 mm, 10 μm). The gradient was 25:75 CH_3_CN/H_2_O to 35:65 CH_3_CN/H_2_O over 20 min for FRIP1 and 20:80 CH_3_CN/H_2_O to 40:60 CH_3_CN/H_2_O over 20 min for FRIP2.

### PET/CT Imaging with ^64^Cu-FRIP1 and ^64^Cu-FRIP2

Approximately 300–400 μCi of ^64^Cu-FRIP1 or ^64^Cu-FRIP2 was intravenously injected via the tail vein into mice bearing FaDu or U87 tumors (n = 4). Mice were imaged using a microPET/CT scanner (Mediso nanoScan PET/CT) at selected time points postinjection. Each static scan consisted of 20 min of PET acquisition followed by a 10 min CT acquisition. PET/CT scans were analyzed using AMIDE (amide.sourceforge.net). The automated SUV calculation tool was used with decay-corrected injected activity and the animal’s body weight. For each volume of interest, a spherical VOI (2–3 mm diameter) was drawn, and SUV values were obtained using VOI statistics.

### Data Analysis and Statistical Considerations for Radioactivity Measurements

All *in vivo* PET data were analyzed using open-source Amide software (amide.sourceforge.net). An unpaired, two-tailed Student’s *t* test was performed in Prism 10 to determine statistically significant differences between two treatment arms in PET imaging and efficacy studies. Statistical significance for Kaplan–Meier survival curves was evaluated using the Mantel-Cox test. For all analysis, *p* < 0.05 was considered statistically significant (*: *p* < 0.05, **: *p* < 0.01, ***: *p* < 0.001)

### Antitumor Assessment Studies with FRIP1-VCMMAE

The study was divided into 3 arms (n = 8/arm): saline, FRIP1-VCMMAE (2 mg/kg per injection, dissolved in saline with 1% DMSO), and MMAE (0.3 mg/kg per injection, dissolved in saline with 1% DMSO). Mice received either saline, FRIP1-VCMMAE, or MMAE via IP injection 3 times per week for 3 weeks. Mice were monitored for tumor sizes and body weight every other day. Mice were euthanized if they reached the experimental endpoint of tumor volume >2000 mm^3^, ≥ 15% loss in mouse body weight or tumor ulceration >50% of tumor surface.

### Antitumor Assessment Studies with ^67^Cu-FRIP2

The study was divided into 2 arms (n = 8/arm): saline and ^67^Cu-FRIP2. Mice received either saline or a single dose of 1 mCi ^67^Cu-FRIP2 on Day 0. Mice were monitored for tumor volume and body weight every other day. Mice were euthanized if they reached experimental end point of tumor volume >2000 mm^3^, ≥ 15% loss in mouse body weight or tumor ulceration >50% of tumor surface.

## Supplementary Material





## Notes

IceLogo analysis; top 20 sequences from MSP-MS optimization; Western blot analysis of FAP expression in cell lines; fixed-cell confocal imaging; UAMC-1110 inhibition assay; MTT assay with UAMC-1110 in U-251 cells; live-cell confocal imaging (0, 4, and 8 min); membrane disruption by FRIP1-Cy5; MTT assays of FRIP1 and cleaved FRIP1 in U-251 cells; MTT assays of Cys-VCMMAE and VCMMAE in U-251 cells; Western blot analysis of modulated FAP expression; synthesis and characterization of IR780; protease activity assays with human FAP, mouse FAP, and human DPP4; anti-FAP immunohistochemistry of FaDu tumors; in vivo imaging of FRIP1-IR780; in vivo efficacy studies (vehicle, FRIP1-VCMMAE, and MMAE); survival analysis of treated FaDu tumor-bearing mice; HPLC analysis of FAP-mediated cleavage of ^64^Cu-FRIP1; molecular structures and analytical characterization (HPLC and LC–MS) of compounds used in this study (PDF)
Supporting Videos S1–S3, live-cell confocal imaging of FRIP1-Cy5 cellular association (ZIP)
