# Supplementary material for: A Prodrug Strategy to Conditionally Trap Therapeutic Payloads for Improved Tumor Retention
Source: ACS Cent Sci. 2026 May 13;12(5):719–30. doi: 10.1021/acscentsci.6c00185 (PMC13220212; doi:10.1021/acscentsci.6c00185)
Supplement: Supplementary file 1 [file oc6c00185_si_001.pdf]

Supporting Information for

A prodrug strategy to conditionally trap therapeutic payloads for improved tumor retention

Deokhee Kang[a†], Apurva Pandey[b†], Garima Kumar[b], Abijeet S. Mehta[c], Tyler C. Detomasi[a], Dashiell Anderson[a], Conner Bardine[a], Garrison Asper[a], Junyang Qi[b], Isha Nadig[d,e], Yifan Cui[b], Fiona M. Quimby[b], Jesse Ling[b], Youngho Seo[b], Bruce E. Cohen[d,f], Mekhail Anwar[c], Michael J. Evans[b*], Charles S. Craik[a*]

[a] Department of Pharmaceutical Chemistry, University of California, San Francisco, San Francisco, California, 94143, United States.
[b] Department of Radiology and Biomedical Imaging, University of California, San Francisco, San Francisco, California, 94158, United States.
[c] Department of Radiation Oncology, University of California, San Francisco, San Francisco, California, 94158, United States.
[d] The Molecular Foundry, Lawrence Berkeley National Laboratory, Berkeley, California, 94720, United States.
[e] Department of Chemistry, University of California, Berkeley, California, 94720, United States.
[f] Division of Molecular Biophysics & Integrated Bioimaging, Lawrence Berkeley National Laboratory, Berkeley, California, 94720, United States.

[†] These authors contributed equally to this work.

* Email: charles.craik@ucsf.edu; michael.evans@ucsf.edu

Supplementary Figures S2
Appendix: Synthesis of Molecules Used in This Study S21
Legends for Supplementary Videos S28

Supplementary Figures

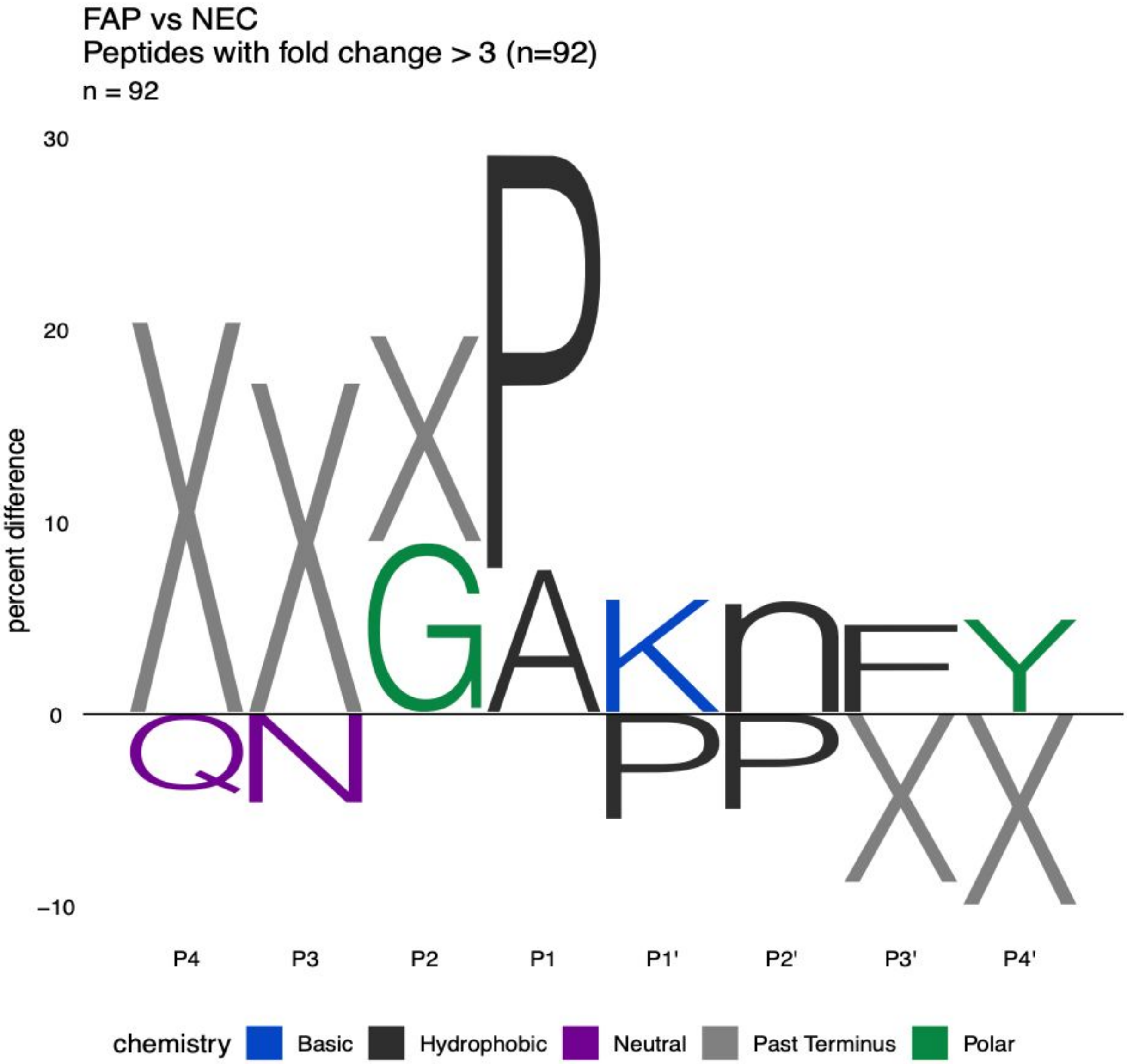

Supplementary Figure 1. An IceLogo plot built out of the MSP-MS for FAP.

P4	P3	P2	P1	P1’	P2’	P3’	P4’	Log_2(Fold Change)
**H**	**I**	**G**	**P**	**T**	**A**	**A**	**Y**	9.947
P	T	G	P	n	X	X	X	8.809
X	S	G	P	D	Y	Q	K	8.408
D	R	G	P	W	R	F	X	8.390
**H**	**Q**	**G**	**P**	**F**	**W**	**n**	**L**	7.479
I	n	G	P	D	A	F	N	6.533
n	H	S	P	W	T	n	A	6.316
X	X	N	P	T	S	T	Y	5.949
Y	H	G	P	L	A	H	X	5.765
H	Y	G	P	T	V	N	K	5.132
G	n	G	P	F	H	I	V	4.963
A	R	Q	P	W	N	n	L	4.750
S	L	S	K	n	N	P	V	4.621
F	Y	T	S	Q	I	P	A	4.365
H	L	A	G	K	R	R	D	4.304
X	X	T	W	R	G	V	S	3.874
H	D	F	G	V	W	K	A	3.831
X	X	G	P	K	L	T	Y	3.758
X	X	F	R	I	H	G	F	3.725
A	R	S	A	F	A	E	n	3.723

n: norleucine
X: past terminus

Supplementary Figure 2. Top 20 FAP-cleavable sequences identified from MSP-MS optimization. As previously reported, proline was strongly preferred at the P1 site, while glycine was favorably enriched at the P2 site. The top two 8-mer peptide sequences were selected and designated as FRIP1 and FRIP2, respectively.

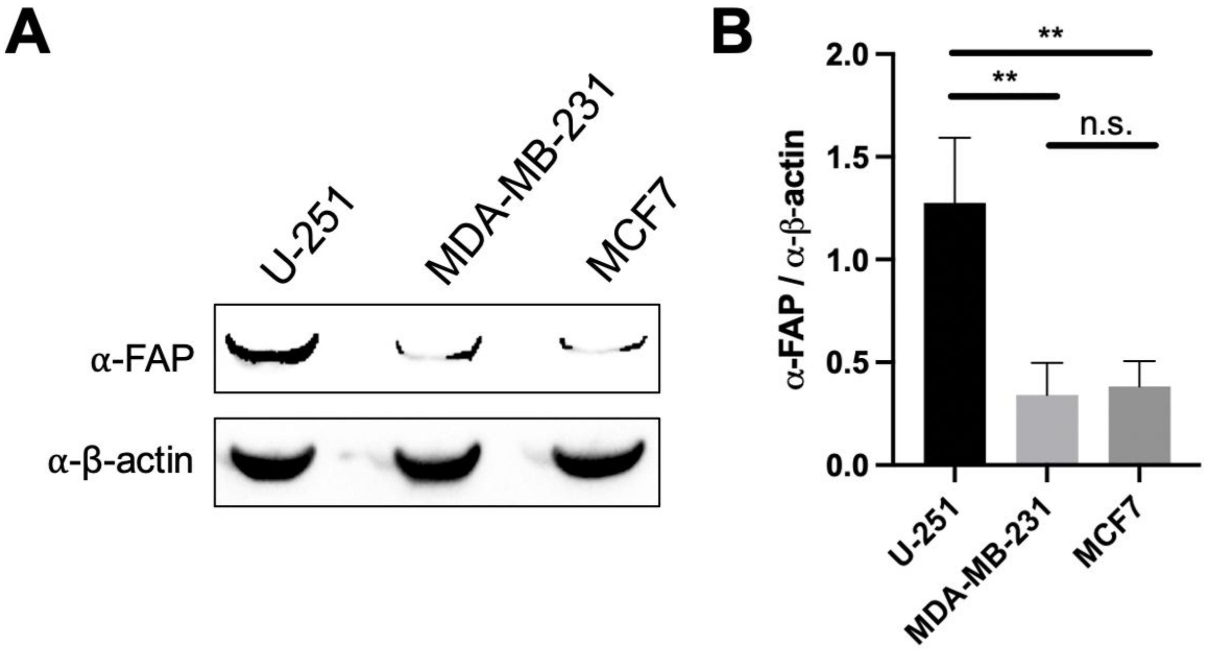

Supplementary Figure 3. Western blot analysis comparing FAP expression levels among U-251, MDA-MB-231, and MCF7 cancer cell lines. (A) Western blot analysis of homogenates from U-251, MDA-MB-231, and MCF7 cell lines. Anti-FAP antibody was used to detect FAP expression, with anti-β-actin as a loading control. The glioblastoma U-251 cell line showed the highest FAP expression. (B) Quantification of Western blot results. (*: P<0.05, **: P<0.01, ***: P<0.001, n.s.: not significant; N=3)

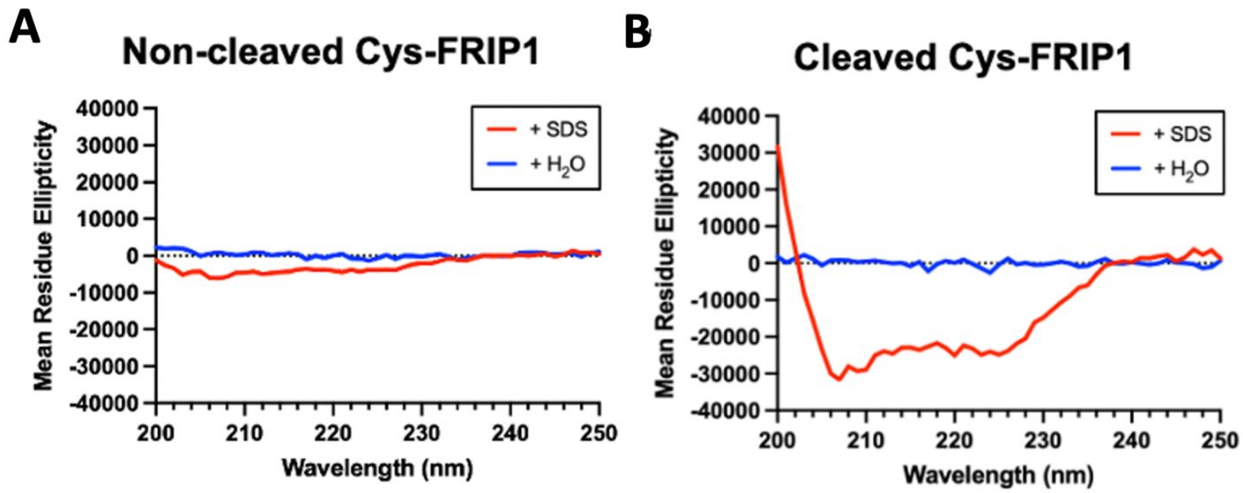

Supplementary Figure 4. Circular dichroism (CD) analysis of Cys-FRIP1 secondary structure. (A) Non-cleaved Cys-FRIP1 exhibits minimal alpha helical content in the presence of membrane-mimicking SDS micelles. (B) Cleaved Cys-FRIP1 shows a pronounced increase in alpha helical content under the same conditions, consistent with membrane-induced helix formation.

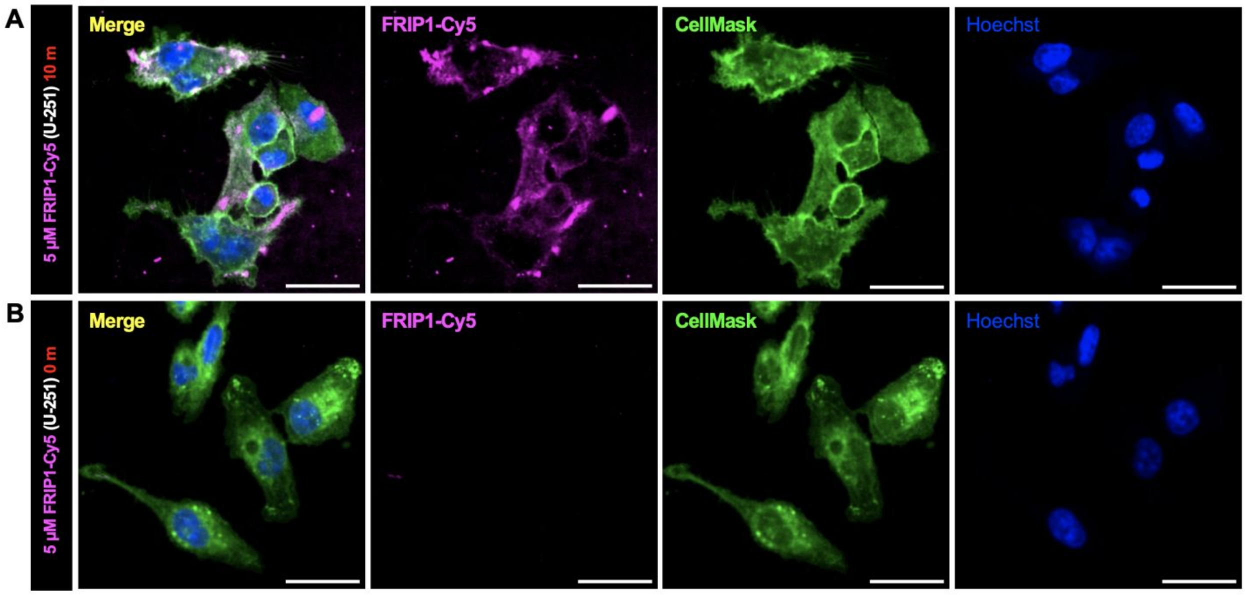

Supplementary Figure 5. Membrane interaction and internalization of FRIP1-Cy5. (A) Confocal micrographs of U-251 cells 10 min after treatment with 5 µM FRIP1-Cy5. FRIP1-Cy5 was observed at the cell membrane and diffused into the cytoplasm. (B) Confocal micrographs of U-251 cells without incubation with 5 µM FRIP1-Cy5. A brief pulse of FRIP1-Cy5 did not allow the catalytic process to occur, resulting in no detectable FRIP1-Cy5 fluorescence in the cells (magenta: FRIP1-Cy5, green: CellMask Green, blue: Hoechst 33342, scale bar: 20 µm).

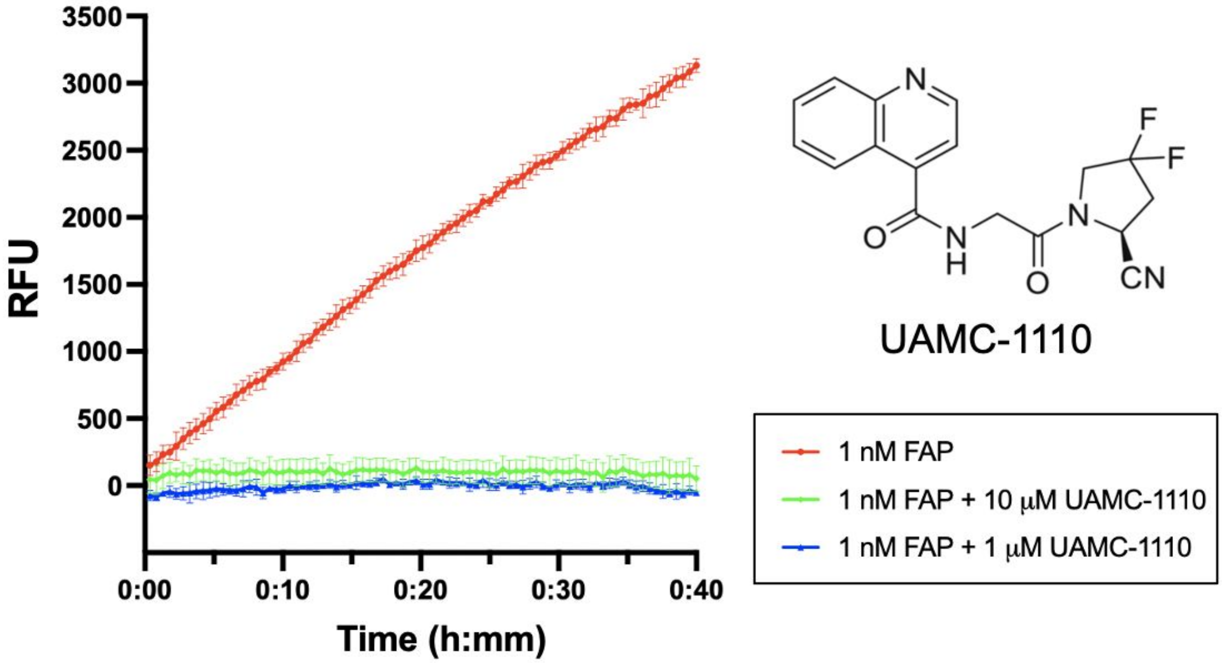

Supplementary Figure 6. Inhibitory effects of UAMC-1110 on FAP activity. Both 1 μM and 10 μM concentrations of the FAP-specific inhibitor UAMC-1110 were added to 1 nM FAP, resulting in complete inhibition of enzymatic activity.

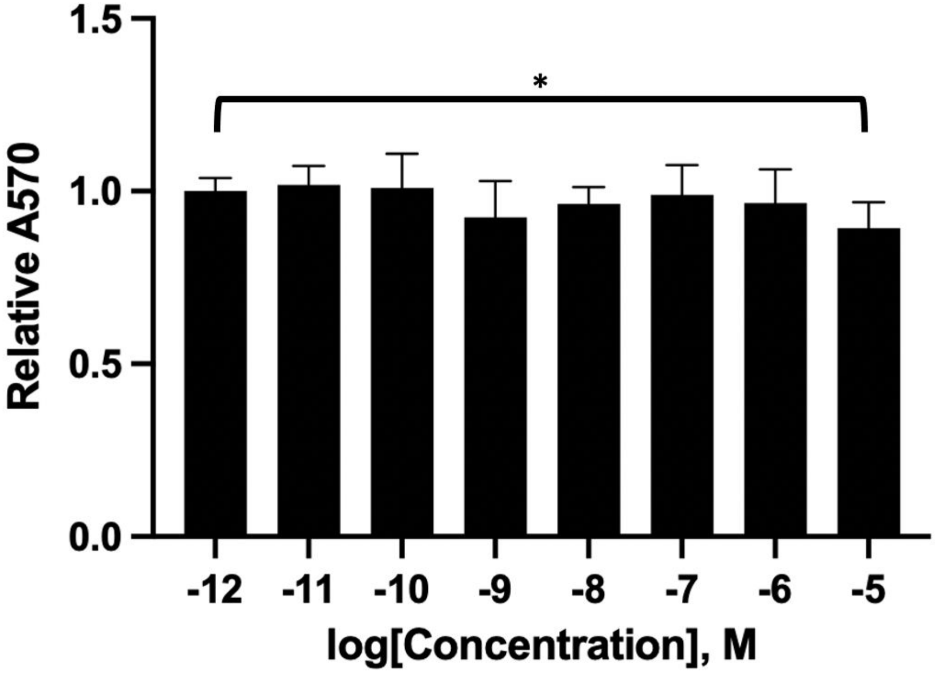

Supplementary Figure 7. Cytotoxicity of UAMC-1110. Treatment of U-251 cells with 10 μM UAMC-1110 resulted in mild toxicity, as assessed by MTT assay.

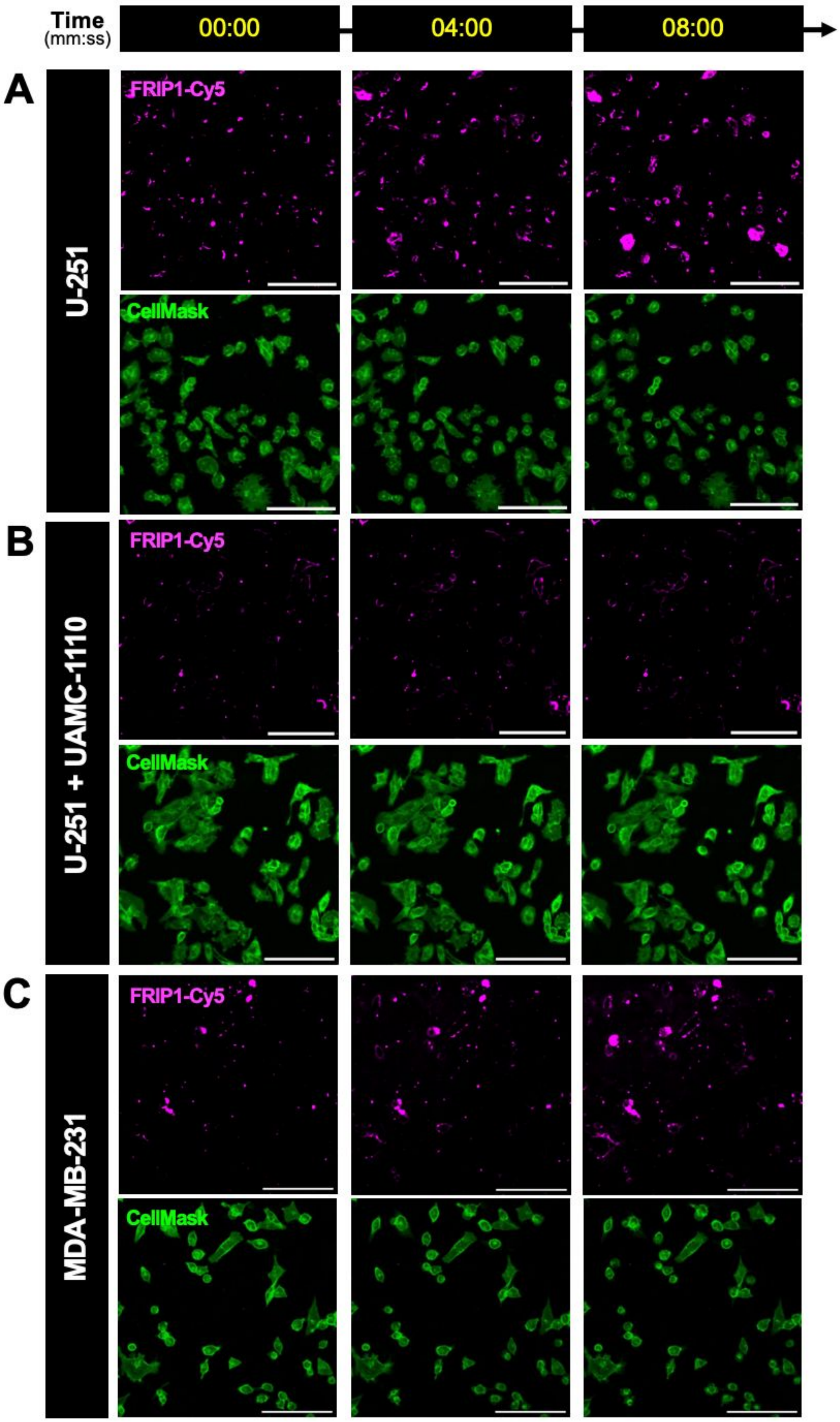
Time
(mm:ss)
00:00
04:00
08:00
A
U-251
FRIP1-Cy5
CellMask
B
U-251 + UAMC-1110
FRIP1-Cy5
CellMask
C
MDA-MB-231
FRIP1-Cy5
CellMask

Supplementary Figure 8. FAP-dependent membrane interaction and internalization of FRIP1-Cy5. (A) FRIP1-Cy5 progressively accumulated on the cell membrane and was internalized in U-251 cells over time. (B) Treatment with 1 μM UAMC-1110 markedly reduced FRIP1-Cy5 membrane engagement and internalization. (C) MDA-MB-231 cells, which express lower levels of FAP, also showed increasing FRIP1-Cy5 fluorescence, but to a lesser extent than U-251 cells with higher FAP expression (magenta: FRIP1-Cy5, green: CellMask Green, scale bar: 100 μm).

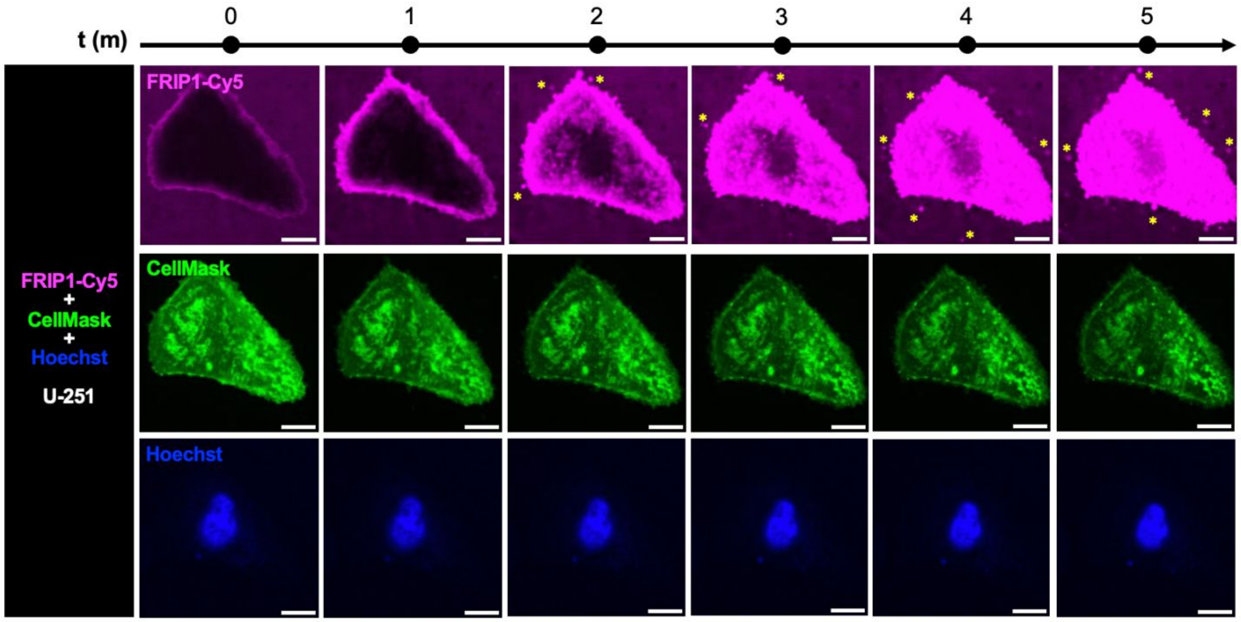

Supplementary Figure 9. Generation of FRIP1-Cy5 clusters following cell membrane rupture. A subset of cancer cells exhibited rapid accumulation of FRIP1-Cy5 (magenta) at the membrane, leading to membrane rupture and the subsequent formation and dispersion of FRIP1-Cy5 puncta (indicated by yellow asterisks). Cell membranes were outlined with CellMask Green (green), and nuclei were labeled with Hoechst 33342 (blue) (scale bar: 10 μm).

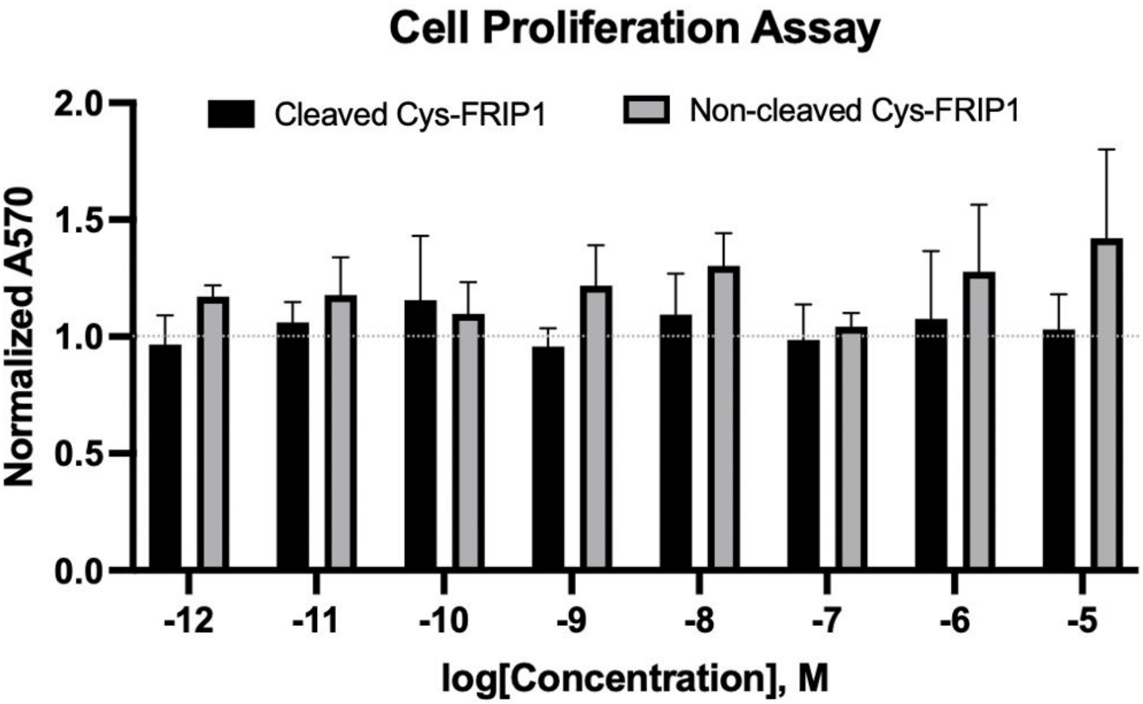

Supplementary Figure 10. Cytotoxicity of Cys-FRIP1 and cleaved Cys-FRIP1. An MTT assay was performed on U-251 cells to evaluate the intrinsic toxicity of Cys-FRIP1. Across the tested concentration range (1 pM to 10 μM), neither Cys-FRIP1 nor its cleaved form exhibited detectable cytotoxic effects.

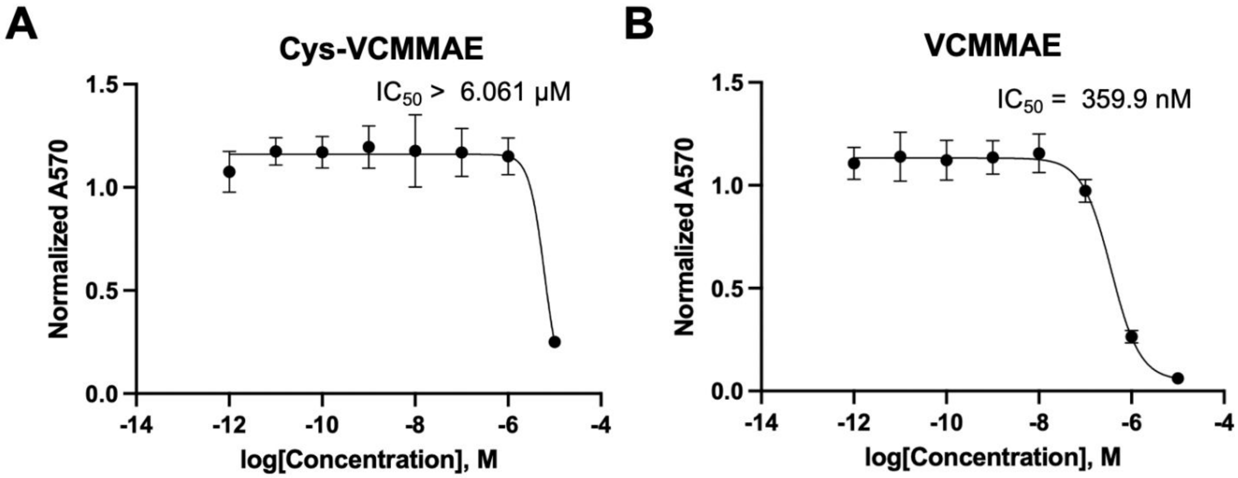

Supplementary Figure 11. Cytotoxicity of Cys-VCMMAE and VCMMAE on U-251 cells. An MTT assay was performed on U-251 cells to evaluate the cytotoxic effects of (A) Cys-VCMMAE and (B) VCMMAE.

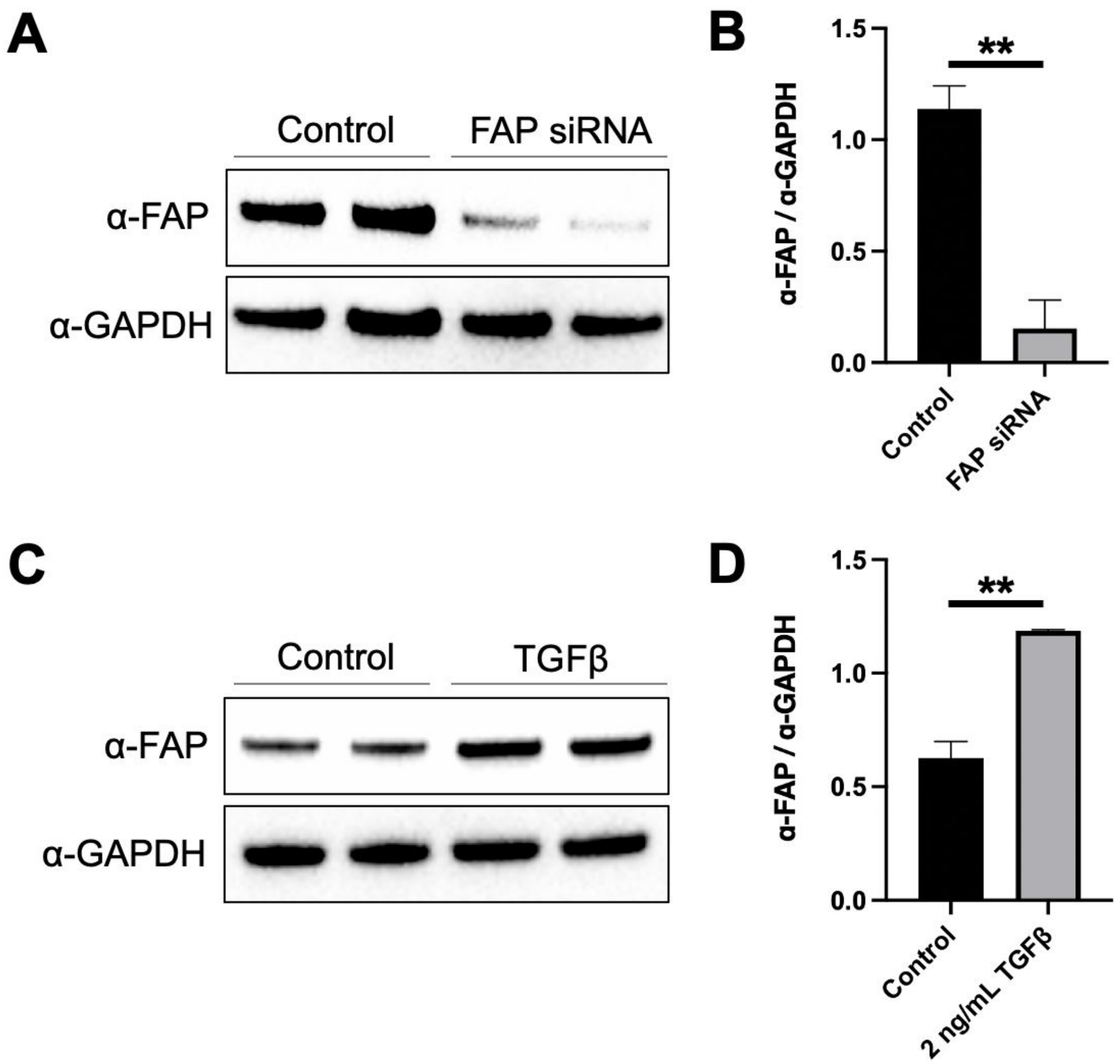

Supplementary Figure 12. Modulation of FAP expression levels in U-251 cells. (A) Western blot showing FAP knockdown following treatment with FAP siRNA. (B) Quantification of FAP knockdown. (C) Western blot showing increased FAP expression upon TGFβ treatment. (D) Quantification of FAP upregulation by TGFβ. (*: P<0.05, **: P<0.01, ***: P<0.001; N=2)

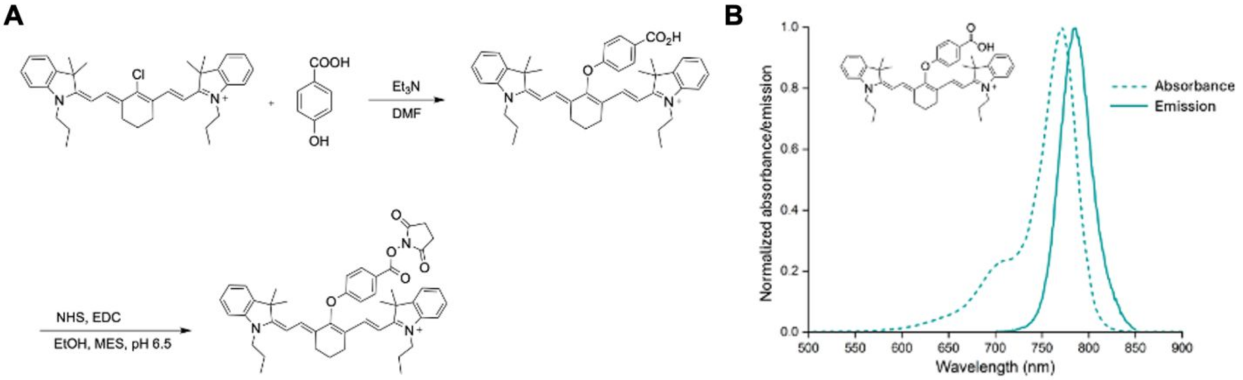

Supplementary Figure 13. Synthesis and spectra of amine-reactive IR780. (A) The near infrared fluorophore IR780-Cl was functionalized with a succinimidyl ester for coupling to the N-terminal amine of FRIP1. (B) Absorbance and emission spectra of IR780.

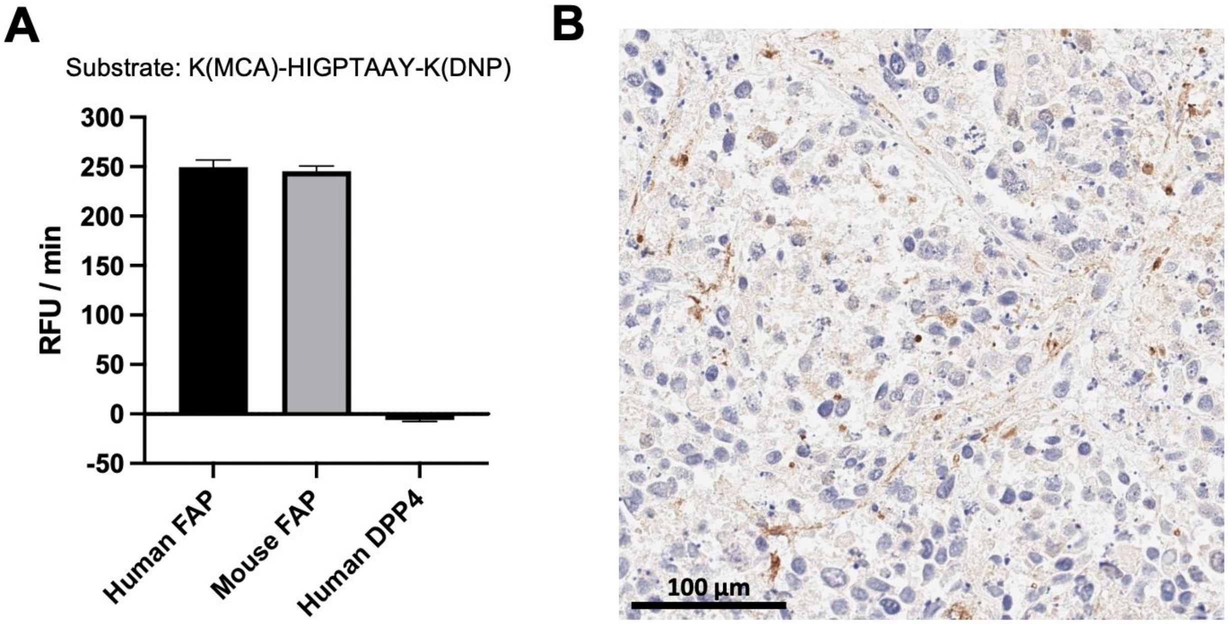

Supplementary Figure 14. Validation of the FaDu-implanted mouse model. (A) Enzymatic activity assay of human FAP, mouse FAP, and human DPP4 using the internally quenched fluorogenic form of FRIP1. Mouse FAP was capable of cleaving the substrate, whereas the closest FAP homolog, DPP4, showed no cleavage activity. (B) Immunohistochemistry analysis of the implanted FaDu tumor using an anti-FAP antibody, confirming FAP expression.

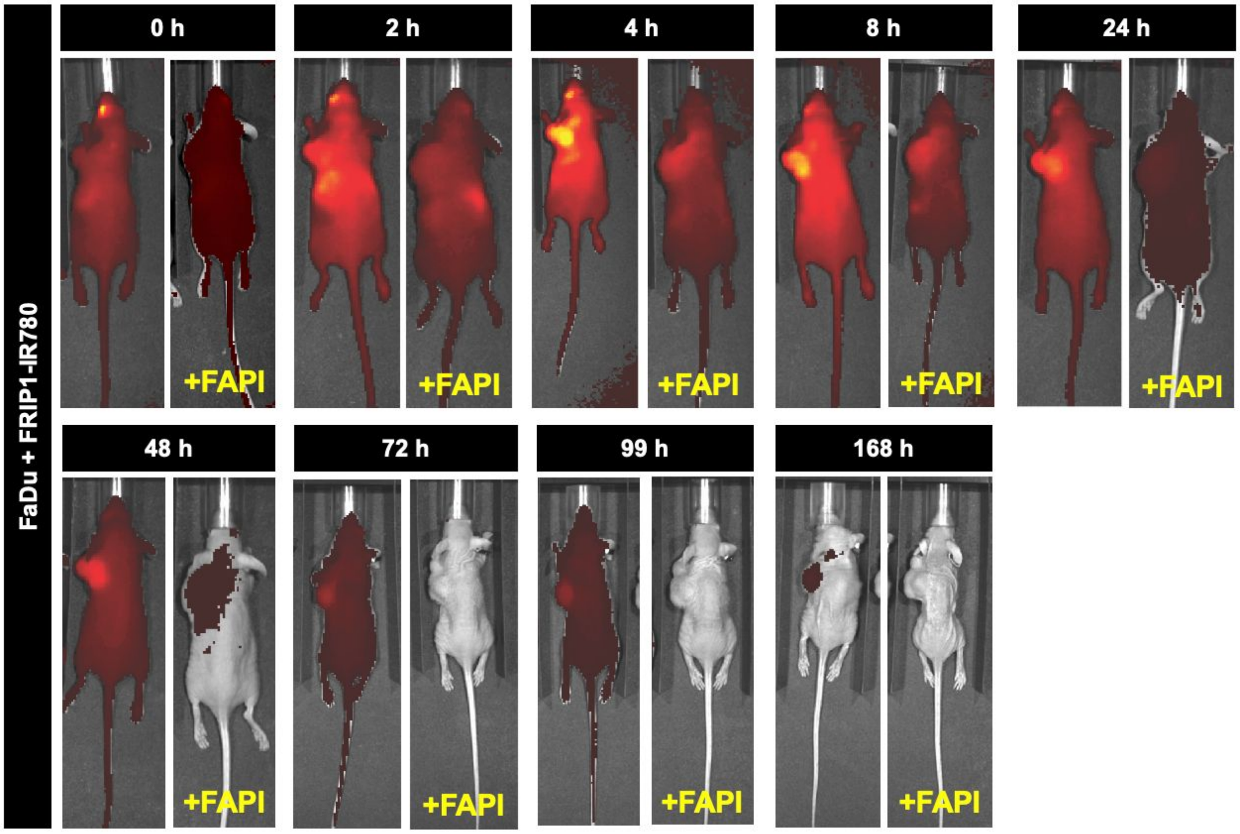

Supplementary Figure 15. Extended *In vivo* biodistribution analysis using FRIP1-IR780. FaDu-implanted mice pre-injected with FAPI (UAMC-1110) exhibited reduced tumoral uptake of FRIP1-IR780 throughout the 7-day monitoring period.

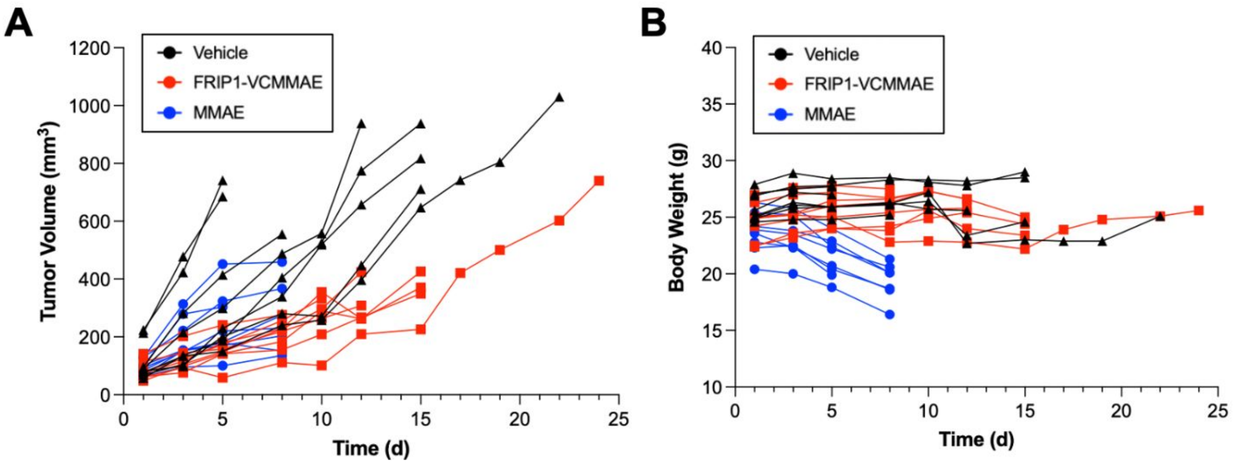

Supplementary Figure 16. Individual tumor volumes and body weights of FaDu-implanted mice treated with vehicle, FRIP1-VCMMAE, or MMAE. (A) A cohort treated with FRIP1-VCMMAE showed significantly lower tumor volumes through the 24-day time-course period. (B) Free MMAE cohort exhibited severe weight loss and were euthanized on day 8, while FRIP1-VCMMAE cohort did not show any significant differences from vehicle control group.

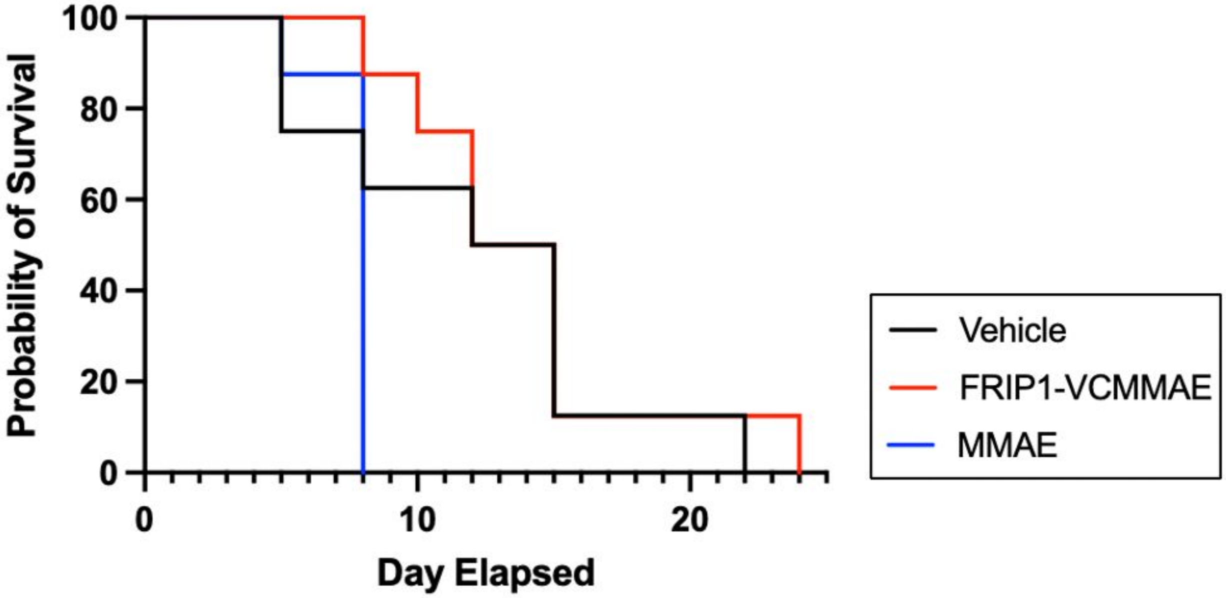

Supplementary Figure 17. Survival plot of FaDu-implanted mice treated with vehicle, FRIP1-VCMMAE, or MMAE. Treatment with FRIP1-VCMMAE moderately increased survival probability compared to the vehicle group. The MMAE cohort reached an early endpoint due to severe weight loss.

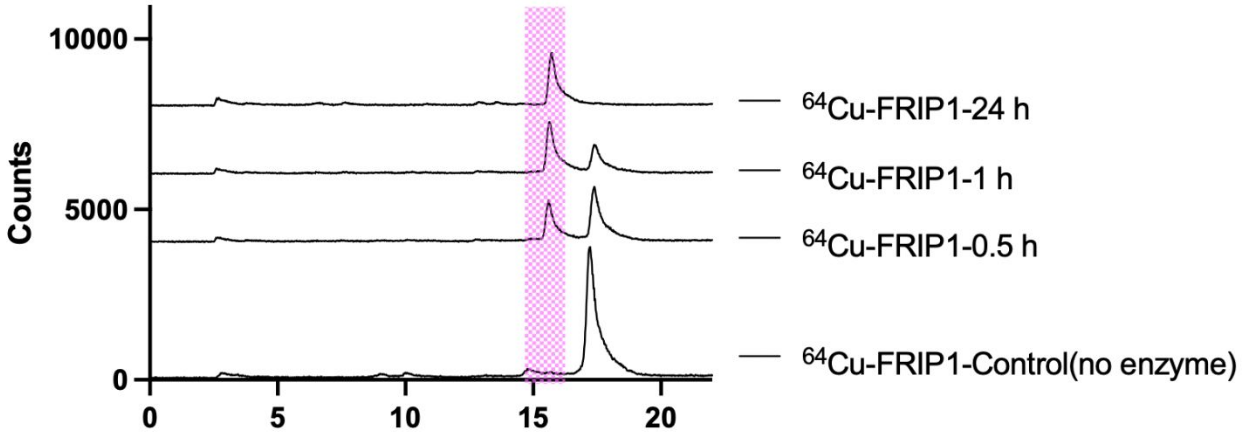

Supplementary Figure 18. FAP cleavage assay of ^{64}Cu-FRIP1. Representative rad-HPLC data showing the conversion of ^{64}Cu-FRIP1 to a single rad peak that aligns with the retention time of the cleaved product.

Appendix: Synthesis of Molecules Used in This Study

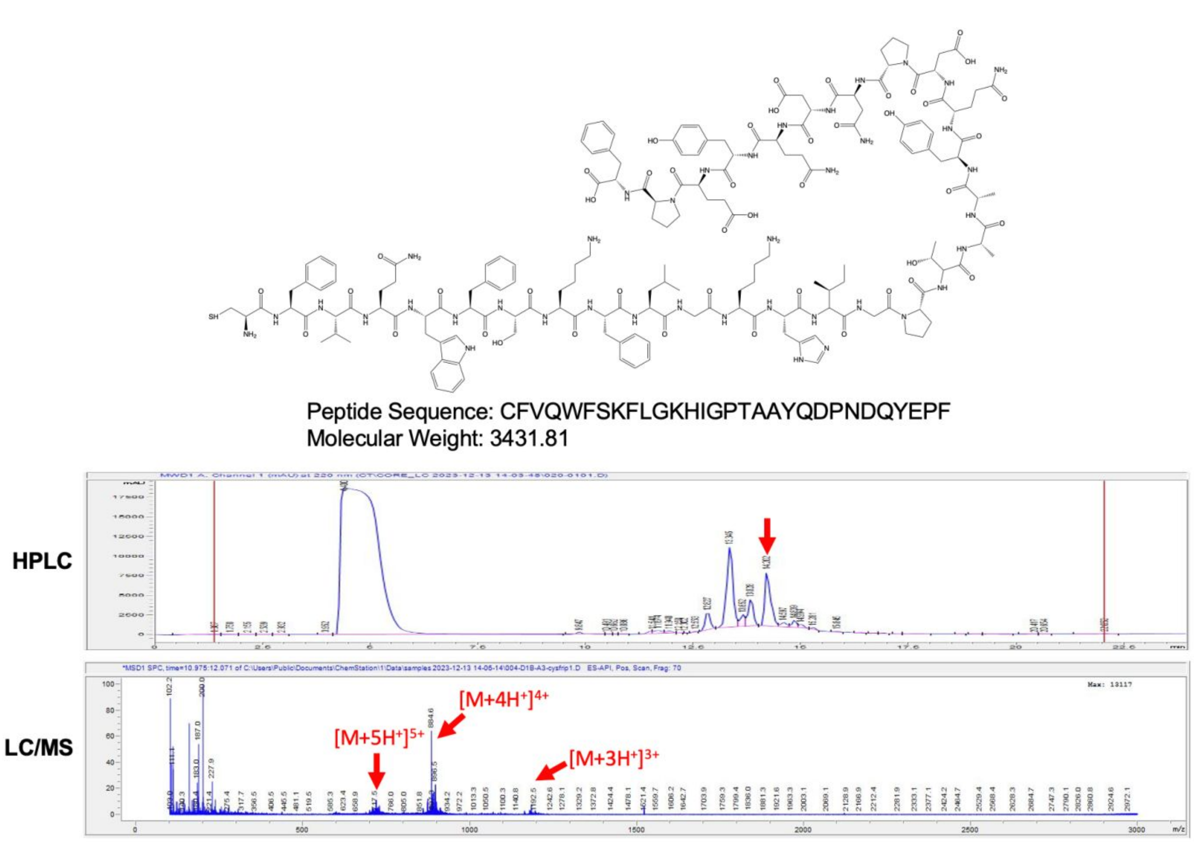

Appendix Figure 1. Synthesis of Cys-FRIP1. The structure, peptide sequence, and molecular weight of Cys-FRIP1 are shown. HPLC and LC/MS spectra confirm the successful synthesis and purity of Cys-FRIP1.

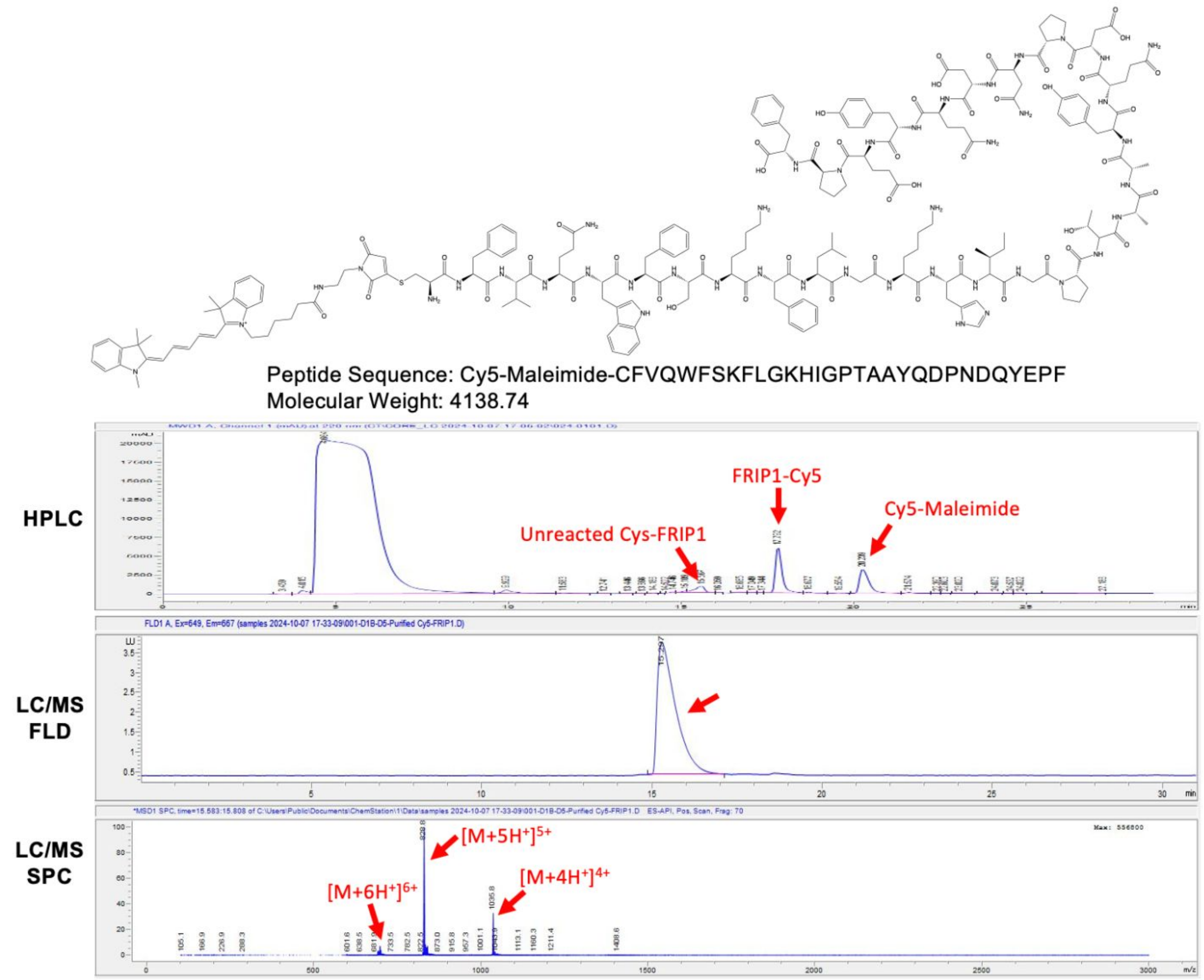

Appendix Figure 2. Synthesis of FRIP1-Cy5. The structure, peptide sequence, and molecular weight of FRIP1-Cy5 are shown. HPLC and LC/MS spectra confirm the successful synthesis and purity of FRIP1-Cy5.

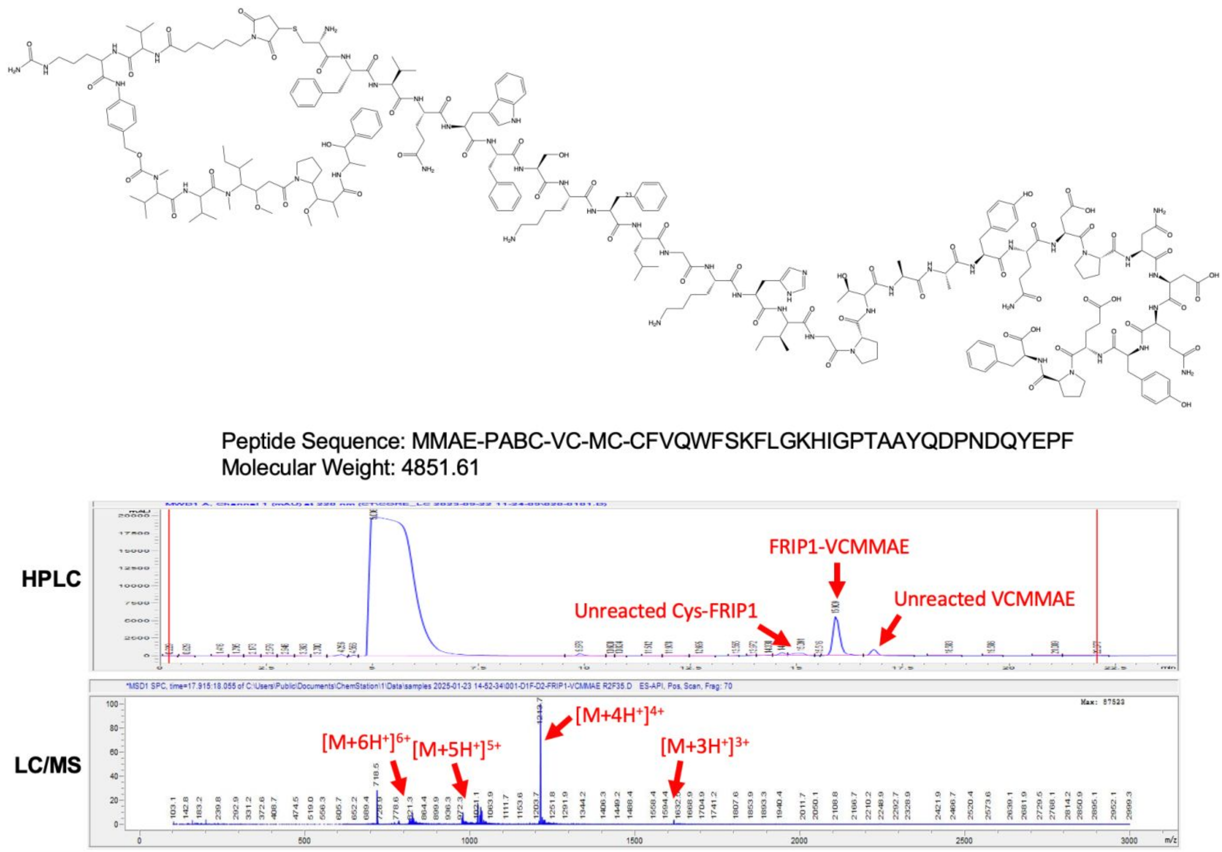

Appendix Figure 3. Synthesis of FRIP1-VCMMAE. The structure, peptide sequence, and molecular weight of FRIP1-VCMMAE are shown. HPLC and LC/MS spectra confirm the successful synthesis and purity of FRIP1-VCMMAE.

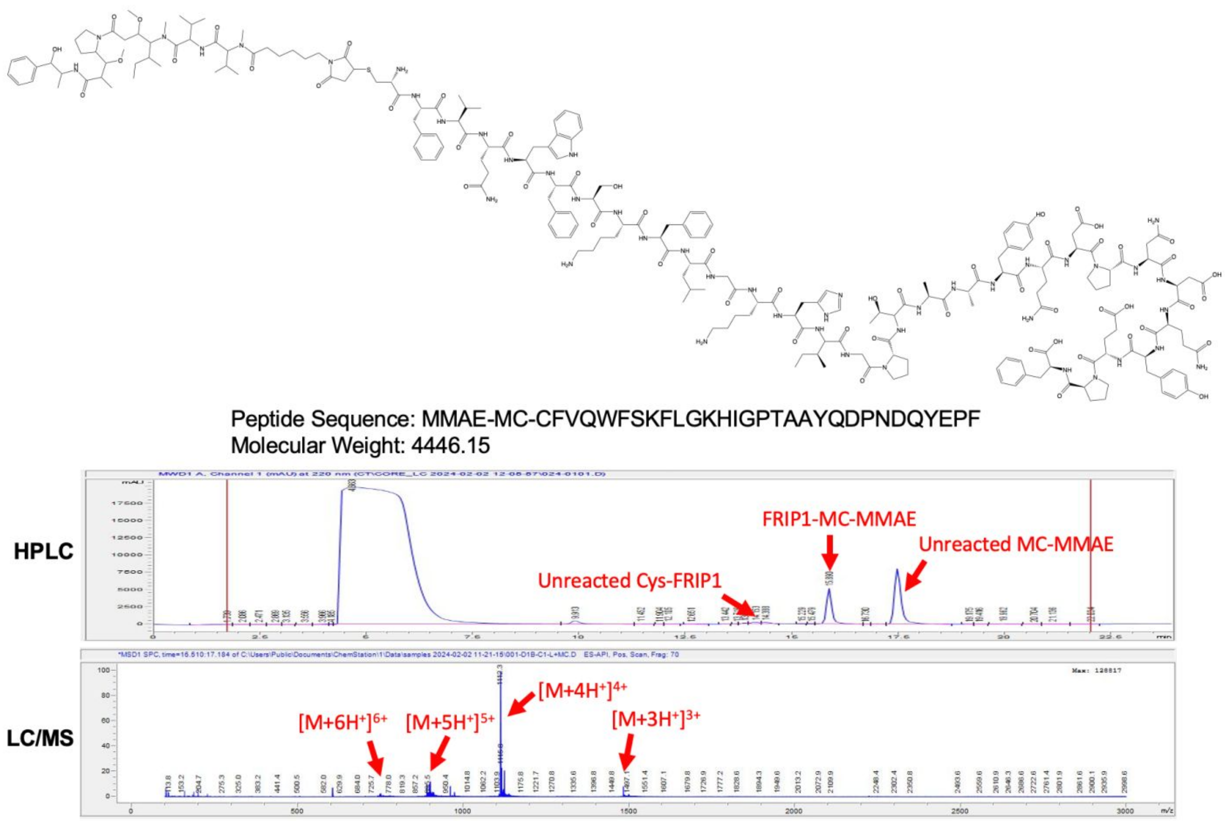

Appendix Figure 4. Synthesis of FRIP1-MC-MMAE. The structure, peptide sequence, and molecular weight of FRIP1-MC-MMAE are shown. HPLC and LC/MS spectra confirm the successful synthesis and purity of FRIP1-MC-MMAE.

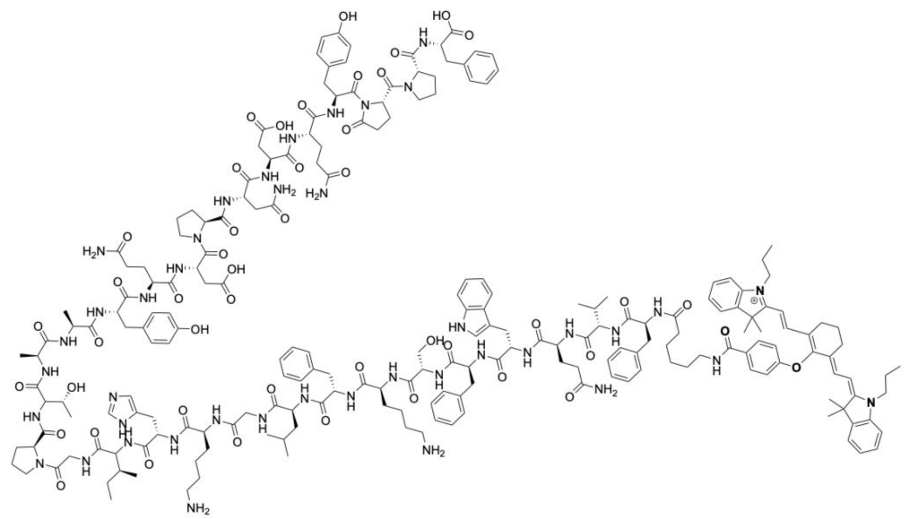

Peptide Sequence: IR780-AHX-FVQWFSKFLGKHIGPTAAYQDPNDQYEPF
Molecular Weight: 4148.08

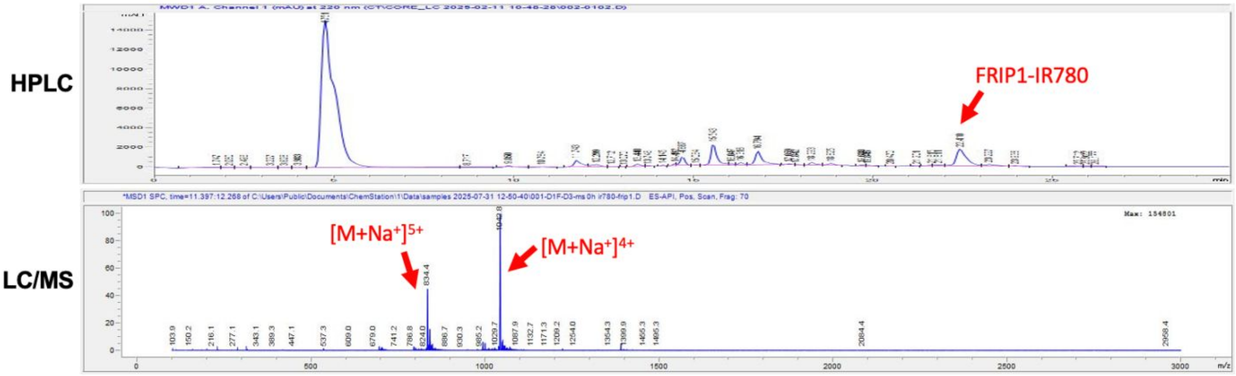

Appendix Figure 5. Synthesis of FRIP1-IR780. The structure, peptide sequence, and molecular weight of FRIP1-IR780 are shown. HPLC and LC/MS spectra confirm the successful synthesis and purity of FRIP1-IR780.

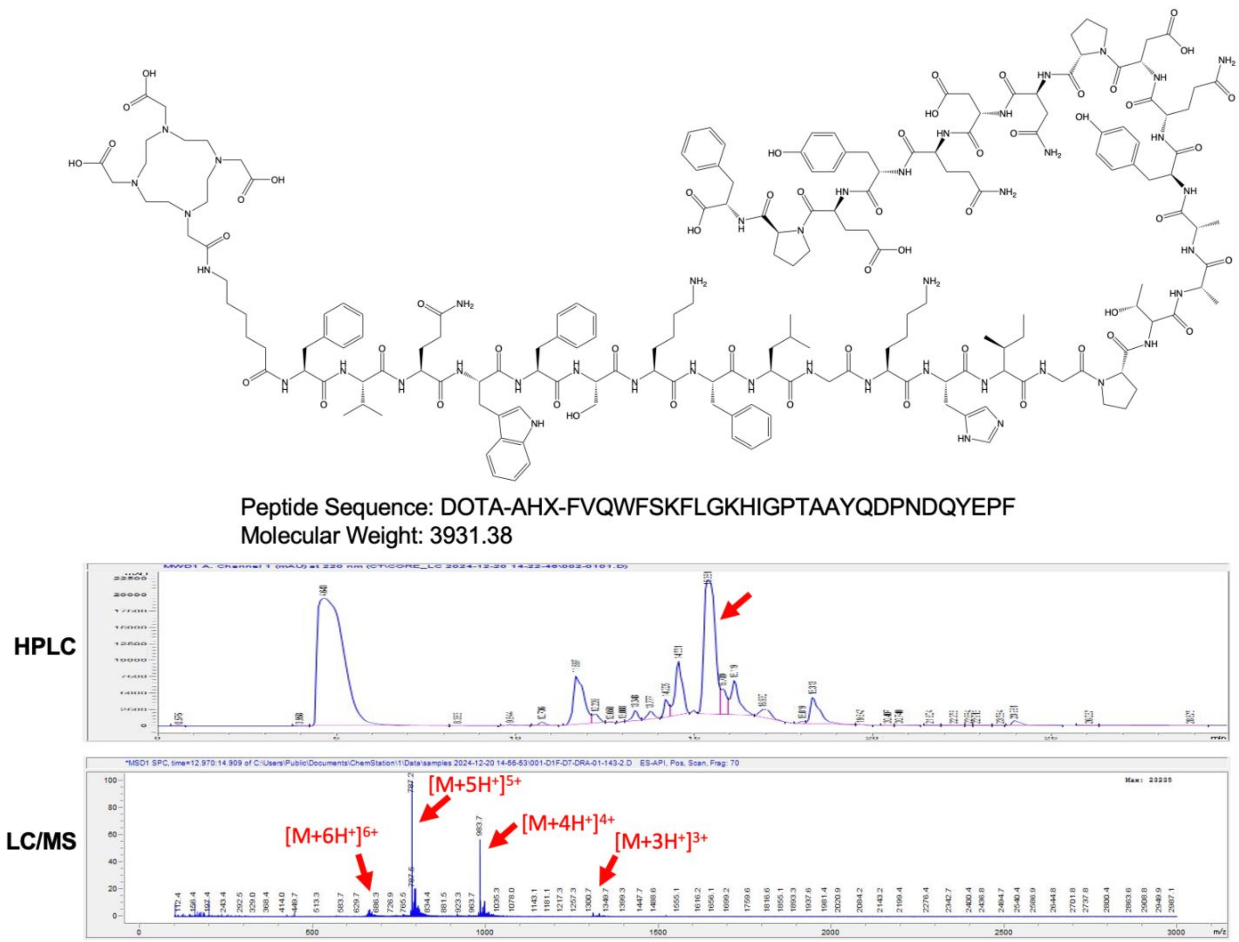

Appendix Figure 6. Synthesis of DOTA-FRIP1. The structure, peptide sequence, and molecular weight of DOTA-FRIP1 are shown. HPLC and LC/MS spectra confirm the successful synthesis and purity of DOTA-FRIP1.

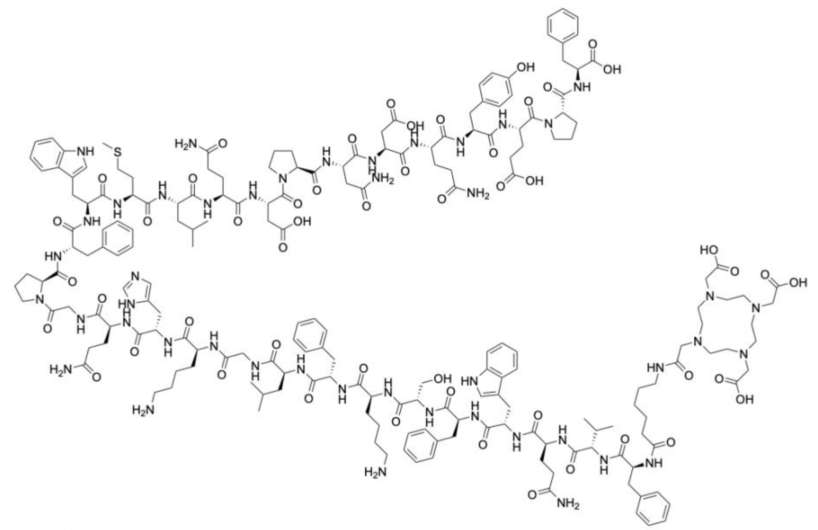

Peptide Sequence: DOTA-AHX-FVQWFSKFLGKHQGPFWMLQDPNDQYEPF
Molecular Weight: 4117.66

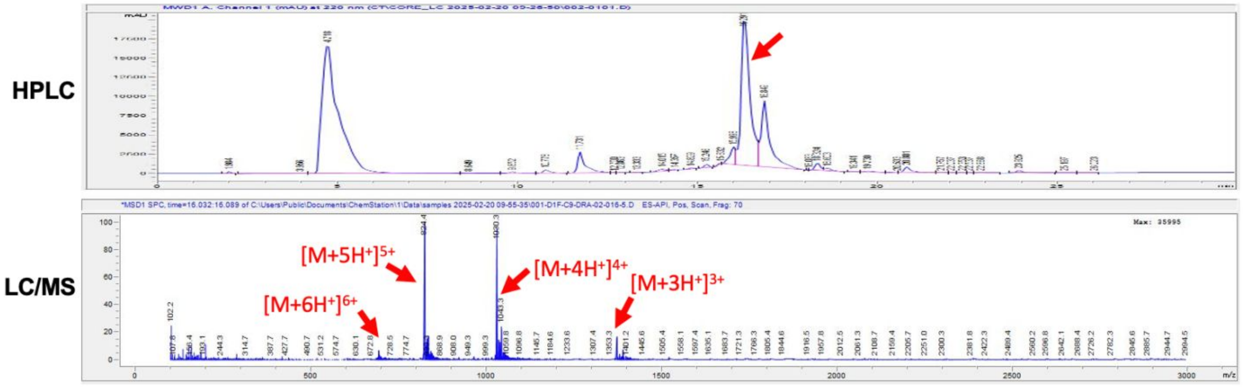

Appendix Figure 7. Synthesis of DOTA-FRIP2. The structure, peptide sequence, and molecular weight of DOTA-FRIP2 are shown. HPLC and LC/MS spectra confirm the successful synthesis and purity of DOTA-FRIP2.

Legends for Supplementary Videos

Supplementary Video 1. Time-lapse confocal imaging of FRIP1-Cy5 in U-251 cells. Cy5 (magenta) accumulated at the cell membrane and was subsequently internalized. Cells were co-stained with CellMask Green (green) to outline the membrane and Hoechst 33342 (blue) to label the nuclei (scale bar: 100 µm). (See the corresponding video file 'Video_S1.avi' provided in the supplementary video folder.)

Supplementary Video 2. Time-lapse confocal imaging of FRIP1-Cy5 in U-251 cells treated with UAMC-1110. Cy5 (magenta) accumulation at the membrane and within the cytosol was markedly reduced in the presence of UAMC-1110. Cells were co-stained with CellMask Green (green) to outline the membrane and Hoechst 33342 (blue) to label the nuclei. (See the corresponding video file 'Video_S2.avi' provided in the supplementary video folder.)

Supplementary Video 3. Time-lapse confocal imaging of FRIP1-Cy5 in MDA-MB-231. Time-lapse confocal imaging of FRIP1-Cy5 in MDA-MB-231 cells. Cy5 (magenta) accumulated at the cell membrane and was subsequently internalized, but at a significantly slower rate compared to U-251 cells. Cells were co-stained with CellMask Green (green) to outline the membrane and Hoechst 33342 (blue) to label the nuclei. (See the corresponding video file 'Video_S3.avi' provided in the supplementary video folder.)
